# Inhibition of cyclin-dependent kinase 8 modulates chondrocyte transition to hypertrophy and provides in vivo protection against spontaneous osteoarthritis

**DOI:** 10.1038/s41413-026-00551-3

**Published:** 2026-08-25

**Authors:** Leah M. Wells, Jacob A. C. Keen, Aikta Sharma, Neil Marr, Rebecca Hansen, Janice S. Pereira, Toby Roe, Helen C. Roberts, Udo Oppermann, Paul A. Clarke, Andrew A. Pitsillides, Scott J. Roberts

**Affiliations:** 1 Skeletal Biology Group, Department of Comparative Biomedical Sciences, The Royal Veterinary College, London, UK; 2 Department of Mechanical Engineering, Faculty of Engineering Sciences, University College London, London, UK; 3 Department of Natural Sciences, Middlesex University, London, UK; 4 Centre for Cancer Drug Discovery, Institute of Cancer Research, London, UK; 5 Botnar Research Centre, Nuffield Department of Orthopaedics, Rheumatology and Musculoskeletal Sciences, University of Oxford, Oxford, UK

**Keywords:** Pathogenesis, Physiology, Diseases, Bone

## Abstract

Chondrocyte hypertrophy is a key hallmark of osteoarthritis (OA) that drives a multitude of whole-joint disease processes. There are no approved disease-modifying pharmaceuticals in clinical use, emphasising the urgent need for innovative therapeutics. Screening of a focussed chemical library using a novel in vitro platform discovered that inhibition of CDK8/19 mediator kinases modulated chondrocyte behaviours with potential to limit OA hallmarks. Transcriptomic analysis of various chondrogenic cell populations revealed that CDK8/19-inhibitor treatment upregulated pro-anabolic/anti-catabolic markers, downregulated markers of hypertrophy and protected from IL1β-induced extracellular matrix degradation. CDK8 was also found to be upregulated in chondrocytes in vitro following IL1β stimulation. This translated to human OA cartilage, where CDK8, MED12 and pSTAT1 were found to co-localise to cartilage lesions. Exposure to CDK8/19-inhibitor modified cell metabolism in hypertrophic chondrocytes and reduced inflammatory processes in THP-1-derived macrophages. CDK8/19-inhibitor treatment of the STR/Ort mouse corrected gait asymmetry and improved treadmill completion rates, whilst suppressing weight gain and serum OA biomarkers. Histological analyses of STR/Ort mouse knee joints revealed that in vivo CDK8/19-inhibitor treatment upregulated chondromodulin-1, matrillin-3 and enhanced proteoglycan deposition in the pericellular matrix of growth plate/articular cartilage. Micro computed tomography (µCT) showed that CDK8/19-inhibitor treatment modified chondrocyte endochondral behaviours to limit the number and density of mineralised growth plate bridges, which otherwise accumulate with age in STR/Ort mice. Collectively, these data highlight CDK8/19 inhibition as a promising disease-modifying strategy for targeting whole-joint disease in OA and, for the first time, show that CDK8/19 inhibition exerts pivotal control of multiple cellular processes that are crucial to endochondral ossification/OA.

## Introduction

Osteoarthritis (OA) is a whole joint disease characterised by degeneration and mineralisation of articular cartilage matrix, subchondral bone sclerosis, osteophytogenesis and synovial inflammation.^1^ Many of these cartilage catabolic and joint pathological events are promoted by endochondral ossification processes involving chondrocyte hypertrophy.^2^ Degeneration and vascularisation of articular cartilage, with inflammatory chondrocyte attributes, indeed facilitates a metabolic shift favouring glycolysis that further exacerbates OA progression.^3,4^ These features are also apparent in hypertrophic growth plate chondrocytes during endochondral ossification.^5^

Despite OA being the commonest form of arthritis, there are no disease-modifying therapeutics and clinical development of single-target approaches, including IL1β and ADAMTS5, has failed despite promising data from preclinical models.^6–8^ It is worth emphasising that the current gold standard OARSI scoring criteria used in such preclinical rodent OA models have almost exclusively focused on articular cartilage lesion expansion without accounting for cell-mediated modulation of extracellular matrix (ECM) parameters, which may reflect other facets of disease modification.^9,10^ Together, these issues highlight the need for a revised approach to discover novel OA therapeutics that display multi-pathway/cellular efficacy and in addition, the need for an alternative complementary preclinical scoring system to reveal these cell-mediated processes.

Recapitulation of endochondral processes by hypertrophic chondrocytes is recognised as a key cell-mediated process in OA joint destruction.^11^ Chondrocyte hypertrophy in OA precipitates modified cartilage metabolism through secretion of multiple factors involved in cartilage breakdown, as in endochondral ossification. This is not, however, a synchronised process across the articular cartilage and therefore likely contributes to all stages of OA progression.^2^ The most explored in vivo model of developmental endochondral ossification is the postnatal growth plate that can be used to identify drivers of chondrocyte hypertrophy for therapeutic exploitation in OA.^11^ Notably, recent OA genome-wide association studies have highlighted cartilage and bone developmental signalling pathways to be the most enriched, conferring an increased OA risk.^12^

Markers of chondrocyte hypertrophy are indeed expressed early in OA, making inhibition of this differentiation process a likely therapeutic paradigm to slow or halt OA progression.^13^ For example, Indian Hedgehog (IHH) expression, a key driver of chondrocyte hypertrophy during developmental bone formation with expression diminished in stable articular chondrocytes, is notably also upregulated in OA cartilage, where it correlates with disease severity; inhibition of IHH via activation of fibroblast growth factor receptor 3 (FGFR3) signalling also serves to alleviate OA progression by impeding hypertrophic differentiation.^14,15^ Moreover, intra-articular administration of Sprifermin (recombinant FGF18; resulting in activation of FGFR3) has proven effective at protecting and/or regenerating cartilage in human clinical trials, further highlighting the therapeutic benefit of targeting chondrocyte hypertrophy in OA.^16^

Cartilage mineralisation is another key endochondral ossification process that is driven by chondrocyte hypertrophy, which serves to initiate cartilage catabolism. Importantly, hypertrophic chondrocytes release an abundance of calcium crystals, which are formed in response to the upregulation of hypertrophic markers such as *RUNX2* and *COL10A1*, the latter of which functions as a scaffold for calcification. Matrix vesicles are commonly found to associate with calcium crystals and are presumed to form via “budding” from specific regions of the hypertrophic chondrocyte plasma membrane.^17^ These vesicles further facilitate matrix mineralisation via intraluminal crystal formation and deposition into the cartilage ECM, which initiates matrix degeneration.^18^ The bone morphogenic protein (BMP) pathway is involved in the regulation of chondrocyte hypertrophy and modulation of mineralisation processes, with the BMP antagonist SMOC2 identified as a negative regulator of calcification and a proposed therapeutic target in OA.^19^ Indeed, inhibition of cartilage calcification processes has been proposed as a therapeutic strategy for OA with calcification inhibitors such as phosphocitrate displaying chondroprotective effects both in vitro and in vivo.^20,21^ Together, these studies indicate that inhibition of chondrocyte hypertrophy and subsequent mineralisation processes would be a valuable therapeutic strategy for OA.

Herein, we exploit similarities observed in developmental endochondral ossification processes and OA articular cartilage, as well as in vitro models that faithfully replicate chondrocyte hypertrophy to uncover novel upstream transcriptional/epigenetic therapeutic targets.^11^ We also employ a spontaneous mouse OA model in which an inherent endochondral defect appears to underpin pathologic cartilage ossification, and propose a complementary scoring system to widen the application of OARSI joint scoring to test their effective target translation.^22^ This strategy led to the identification of the mediator-associated CDK8/19 protein kinases as modulators of endochondral ossification. Notably, CDK8/19 inhibitors (termed CDK8i due to kinase effector function being mediated through CDK8^23^) increased chondrocyte glycosaminoglycan (GAG) assembly and reduced mineral deposition in vitro. Furthermore, we demonstrate via an inhibitory strategy that CDK8 functions as a modulator of endochondral ossification in vivo with disease-modifying therapeutic capacity relevant for OA.

## Results

### CDK8i drives a sustained pro-anabolic, anti-hypertrophic phenotype in ATDC5 micromasses

**Fig. 1:**
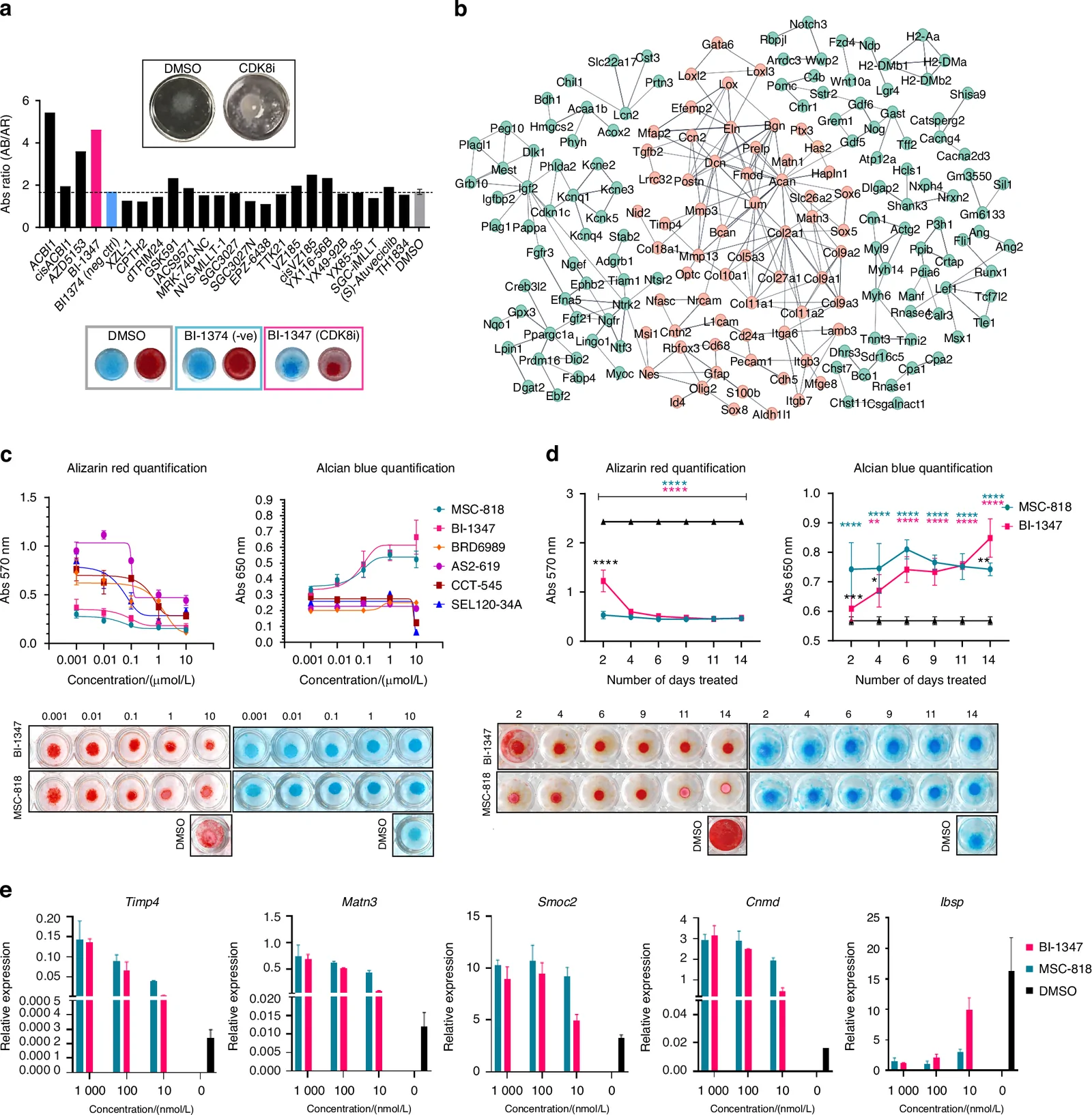
Discovery of CDK8i as a novel modulator of chondrocyte biology in ATDC5 micromasses. **a** Alcian blue (AB):Alizarin red (AR) absorbance ratio identifying CDK8i as a hit (pink). Note effects were absent with the negative control (BI1374; blue), dashed line indicates DMSO control (grey) (initial screen *n* = 1). Cartilage-like CDK8i-treated micromasses (top panel), confirmed by AR:AB staining (bottom panel). **b** STRING interaction network of day 7 upregulated DEGs. Note large network containing ECM components (orange). **c** Mineral deposition (Alizarin red) and GAG deposition (Alcian blue) of Day 14 ATDC5 micromasses treated with various CDK8 inhibitors. (Log(inhibitor) vs. response-variable slope (four parameter) fit; *n* = 3). **d** Phased dosing of ATDC5 micromasses with CDK8i during the 14-day culture period (BI-1347 or MSC-818). Note 48-h exposure is sufficient to maintain phenotypic effects observed with 14 days CDK8i treatment. Significance determined via two-way ANOVA Dunnett’s multiple comparison test. *P ≤ 0.05, **P ≤ 0.01, ***P ≤ 0.001, ****P ≤ 0.000 1 (*n* = 3), teal indicates MSC-818 significance from DMSO, pink indicates BI-1347 significance from DMSO and black indicates MSC-818 significance from BI-1347. **e** Gene expression analysis of day 14 (hypertrophic) ATDC5 micromasses treated with decreasing doses of CDK8i or DMSO. All data *n* = 3, mean ± SD, all measures are significant versus DMSO control (*P* ≤ 0.05). Statistical analysis = two-way ANOVA, Dunnett’s multiple comparison

In vitro screening of an epigenetic/transcriptional targeting compound library^24,25^ using ATDC5 micromass culture revealed several endochondral ossification modulators that maintained or enhanced GAG staining while reducing mineral deposition (Fig. S1). ATDC5-based dose-responses for the 6 top hits, utilising GAG deposition and matrix mineralisation as readouts, and gene expression analysis of chondrogenic (*Sox9, Acan, Col2a1*) and hypertrophic (*Col10a1*) markers prompted selection of the CDK8i, BI-1347,^26^ as the ideal candidate for further interrogation due to promotion of chondrogenic gene expression (Fig. S2 and S3). Treatment with this CDK8i induced micromasses to form a GAG-rich cartilage-like matrix after 7-10 days of treatment with notable and coincident suppression of matrix mineralisation (Fig. 1a).

RNAseq analysis on day 7 CDK8i-treated ATDC5 micromasses identified upregulated differentially regulated genes (DEGs) involved in a highly connected, high confidence STRING network consisting of genes for healthy cartilage ECM (Fig. 1b). Enrichment analysis of upregulated DEGs found the top 10 pathways within biological processes to be related to ECM development and organisation, including connective tissue and cartilage development and ECM organisation (FC: all ~5.0). KEGG pathway enrichment revealed GAG synthesis to be the top upregulated pathway (FC: 7.0), followed by protein metabolism (FC: 6.0), cell adhesion (FC: >3.0) and TGF-beta signalling (FC: >3.0; Fig. S4). RNAseq data also identified multiple genes linked to endochondral ossification and OA, or differentially expressed in healthy cartilage ECM/anti-hypertrophy processes/OA-related proteins (Matrillin-3; *Matn3*, Chondromodulin-1; *Cnmd*, *SPARC* related modular calcium binding-2; *Smoc2* and tissue inhibitor of metalloproteinases-4; *Timp4*) and a promoter of OA mineralisation (integrin bone sialoprotein; *Ibsp*) (Fig. S5-6, supplementary file 2). These were selected as cartilage relevant CDK8i biomarker readouts for subsequent studies.

Dose-response assays (utilising GAG deposition and matrix mineralisation as readouts) using CDK8i of different chemical series (structures in Fig. S7) on ATDC5 micromasses found that the pro-anabolic and mineralisation/hypertrophy-suppressing phenotype promoted by BI-1347 was replicated across all tested inhibitors of CDK8 (Fig. 1c). As MSC2530818 (MSC-818)^27^ induced an identical response to BI-1347, MSC-818 was taken forward alongside BI-1347 for further analysis. Interestingly, flavopiridol, a broad spectrum CDK inhibitor, did not replicate the effects of CDK8i treatment, indicating the chondrogenic effect is at least partly selective to CDK8i (Fig. S8). Phased dosing, whereby ATDC5 micromasses were treated with 1 µmol/L BI-1347 or MSC-818 for 2, 4, 6, 9 or 11 days before switching to control differentiation media (0.1% DMSO) for the remaining culture period (14 days in total), revealed that treatment for only 2 days (MSC-818) at the start of the culture was sufficient to induce changes in GAG matrix deposition and mineralisation which persisted for the entire culture period (Fig. 1d). Thus, 1 μmol/L MSC-818 provoked an increase in GAG deposition (FC: 1.4) and a reduction (FC: 0.2) in mineral deposition at all exposure timeframes (*P* ≤ 0.000 1). Treatment with 1 μmol/L BI-1347 induced a duration-dependent increase in GAG deposition but required at least 4-days of exposure to achieve the same reduction (FC: 0.2) in mineral deposition/Alizarin red staining as MSC-818 (*P* ≤ 0.000 1). To explore whether phenotypic changes identified in RNAseq studies were replicated in hypertrophy-inducing conditions (day 14), gene expression was also assessed at various CDK8i doses (0–1 000 nmol/L; BI-1347 and MSC-818) (Fig. 1e). This showed that CDK8i treatment upregulated expression of several matrix components found in healthy articular cartilage (*Matn3, Timp4, Smoc2* and *Cnmd)* and simultaneously, dose dependently downregulated *Ibsp*. MSC-818 treatment enhanced gene expression changes at lower concentrations (all data *P* ≤ 0.05).

Together, these data suggest that a single 48-h dosing period of CDK8i was sufficient to induce stabilisation of an anabolic/anti-hypertrophic ATDC5 cell phenotype. Furthermore, these data permitted selection of MSC-818 as a more efficacious compound, used for further investigation to probe biology and therapeutic potential of CDK8i for OA. MSC-818 is a highly selective and potent CDK8i, with a biochemical IC_50_ of 2.5 nmol/L against purified CDK8.^27^ Importantly, the ability of MSC-818 to disrupt kinase activity has previously been established using a robust biomarker of CDK8i activity, namely STAT1-S727 phosphorylation.^23^

### Pro-anabolic/anti-catabolic effects of CDK8i are evident in chondrocytes from various species

**Fig. 2:**
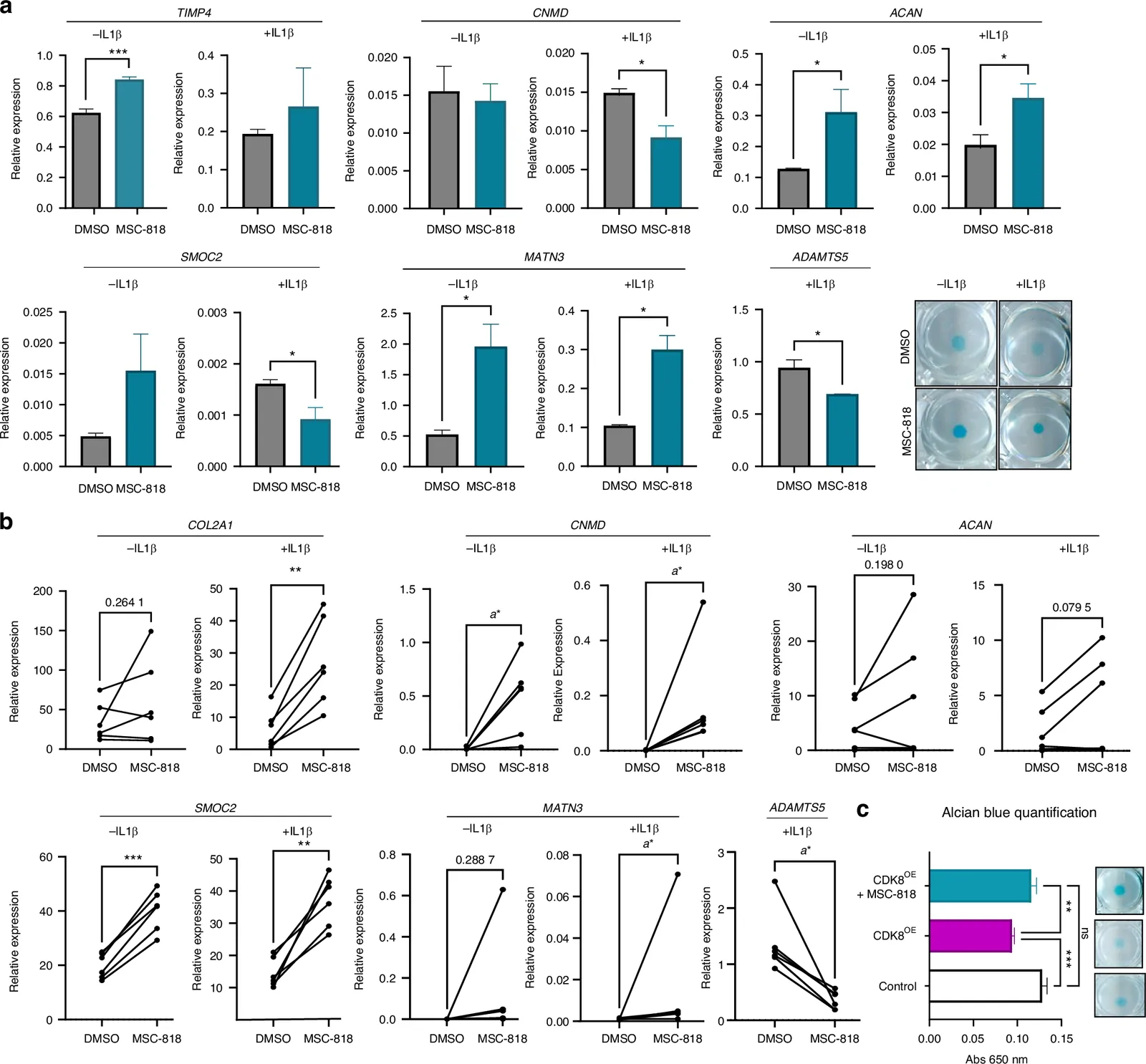
CDK8i protects chondrogenic gene expression under inflammatory conditions across multiple species. **a** Gene expression analysis of CDK8i treated ihMSCs during chondrogenesis (-IL1β) and IL1β OA disease model (+IL1β) conditions. (*N* = 3, error bars = SD, unpaired *t*-test Welch correction). Scanned well images of day 14 ihMSC micromasses treated with 100 nmol/L MSC-818 or DMSO in chondrogenic (-IL1β) or OA disease conditions (+IL1β). **b** Expression analysis of primary equine chondrocytes treated with 100 nmol/L MSC-818 or DMSO control under chondrogenic (-IL1β) or OA disease conditions (+IL1β). *n* = 6 donors, error bars = SD, statistical analysis = paired *t*-test, *a* = Wilcoxon matched-pairs. Interconnected data points represent the average response from an individual equine donor. **c** GAG (Alcian blue) quantification of day 7 control, CDK8 overexpression (OE) and CDK8 OE + 100 nmol/L MSC-818 ihbMSC micromasses. *n* = 3, error bars = SD, statistical analysis = one-way ANOVA, *P ≤ 0.05, **P ≤ 0.01 ***P ≤ 0.001, ****P ≤ 0.000 1

To determine whether the effects of CDK8i in murine ATDC5 cells are replicated in human cells, ihMSCs underwent chondrogenic differentiation in the presence of MSC-818 (or DMSO) before exposure to IL1β in vitro, to mimic OA-like conditions. Under chondrogenic conditions, MSC-818 treatment displayed a pro-anabolic profile in ihMSC-derived chondrocytes with significant increases in *ACAN* (FC: 2.4; *P* ≤ 0.05), *MATN3* (FC: 3.8; *P* ≤ 0.05) and *TIMP4* (FC: 1.4; *P* ≤ 0.001) gene expression. This profile was maintained in the presence of IL1β, with an increase in *ACAN* (FC: 1.8; *P* ≤ 0.05) and *MATN3* (FC: 3.0; *P* ≤ 0.05), whilst reducing *ADAMTS5* expression (FC: 0.8; *P* ≤ 0.05). Interestingly, although hypertrophy was not induced, these cells nonetheless exhibited a reduction in *SMOC2* and *CNMD* (FC: 0.6; *P* ≤ 0.05) (Fig. 2a).

Equine chondrocytes (*n* = 6 donors) were also utilised to underpin these CDK8i effects on primary cell populations and potential utility in horse OA; a major veterinary challenge sharing aetiology with human disease (Fig. 2b). Under chondrogenic culture conditions, MSC-818 significantly enhanced *SMOC2* (FC: 2.0; *P* ≤ 0.001) and *CNMD* (FC: 62.4; *P* ≤ 0.05) gene expression, with a trend toward greater expression in *COL2A1 (P* = 0.26)*, ACAN (P* = 0.20) and *MATN3 (P* = 0.29) compared to DMSO. Again, this pro-anabolic profile was maintained in the presence of IL1β, with significant increases in *COL2A1* (FC*:* 4.3; *P* ≤ 0.01), *MATN3* (FC: 15.3; *P* ≤ 0.05), *SMOC2* (FC: 2.6; *P* ≤ 0.01) and *CNMD* (FC: 84.5; *P* ≤ 0.05), and a trend for increased *ACAN* expression *(P* = 0.08) was observed compared to DMSO. Furthermore, MSC-818 treatment supressed catabolic processes with a reduction in *ADAMTS5* expression (FC: 0.3, *P* ≤ 0.05). These data show that CDK8 inhibition by MSC-818 exerts cross-species anabolic/anti-catabolic gene expression changes in both chondrogenic and OA-like conditions in vitro.

To assess whether CDK8 overexpression reciprocally modifies chondrogenesis, immortalised human bone marrow MSCs (ihbMSC) were modified (lentiviral) with WT CDK8 (CDK8^OE^). Resultant positive cell populations were subjected to chondrogenic differentiation in micromass culture in the presence of MSC-818 for 7 days; DMSO treated control micromasses were used to assess baseline GAG deposition (Fig. 2c). We found that CDK8 overexpression significantly suppressed GAG deposition (FC: 0.7, *P* ≤ 0.001), indicating a restriction of chondrogenic differentiation/anabolic processes. Negative effects of CDK8^OE^ were rescued by CDK8i, with 100 nmol/L MSC-818 treatment restoring GAG deposition (*P* ≤ 0.01) to levels identical to control micromasses (CDK8^OE^ + MSC-818 vs Control; *P* > 0.05).

### CDK8i modifies inflammatory macrophage processes and chondrocyte metabolic profiles

**Fig. 3:**
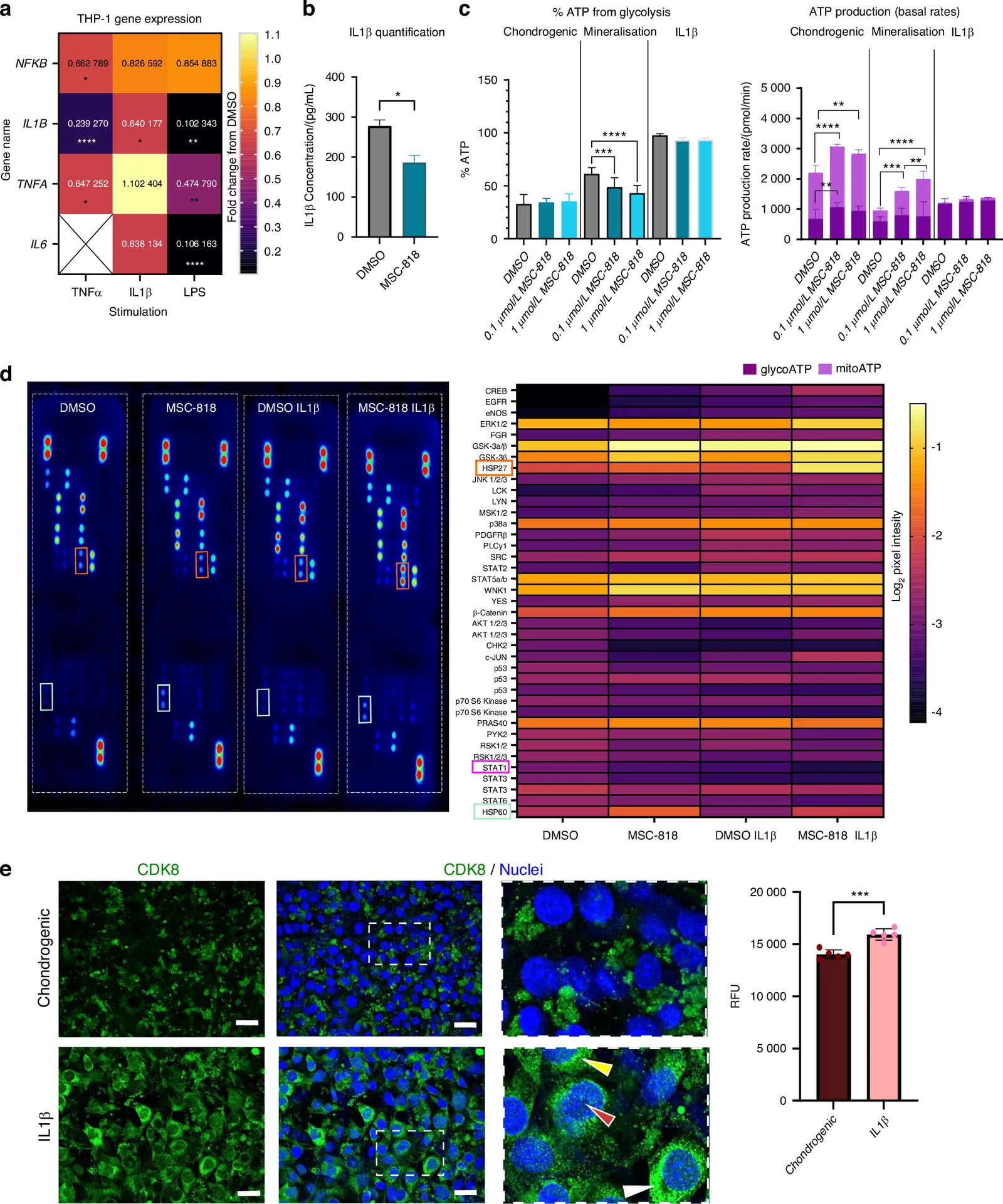
CDK8i in immunometabolism. **a** Heat map gene expression analysis of THP-1-derived macrophages stimulated with inflammatory mediators (LPS, TNFα or IL1β) in the presence of 100 nmol/L MSC-818 or DMSO. Data points show fold change from DMSO. Note TNFα stimulation did not induce IL6 expression (*n* = 3). **b** Secreted IL1β protein quantification of THP-1-derived macrophage conditioned media following LPS stimulation with MSC-818 or DMSO treatment. *P ≤ 0.05, **P ≤ 0.01 ***P ≤ 0.001, ****P ≤ 0.000 1, *n* = 2, statistical analysis = Welch’s *t*-test. **c** MitoStressTest and % ATP produced via glycolysis in MSC-818 (1 μmol/L or 0.1 μmol/L)-treated ATDC5 micromasses ( ~ 6 wells/condition), statistical analysis = two-way ANOVA (Šídák’s and Tukey’s ANOVA, respectively). **P ≤ 0.01 ***P ≤ 0.001, ****P ≤ 0.000 1. **d** Heatmap of phospho-kinase array of ihMSC micromasses under chondrogenic (-IL1β) and OA-like conditions ( + IL1β). Notable effects seen in HSP27 (orange), total HSP60 (green) and p-STAT1 (CDK8 target; pink) with MSC-818. Quantified dot intensities following image analysis (Log_2_ pixel intensity from each blot probed with pooled ihMSC micromasses; 24 wells/condition). **e** CDK8 expression (green) in ATDC5 micromasses under chondrogenic and OA-like ( + IL1β) conditions. Note upregulation of CDK8 and increased nuclear (red), perinuclear (yellow) and cytoplasmic expression (white) following IL1β stimulation. Scale bar = 25 μm. *n* = 5 wells/condition, error bars=SD, statistical analysis = Welch’s *t*-test, ***P ≤ 0.001

As OA is a whole joint disease involving local inflammation, we also assessed the effects of CDK8i (100 nmol/L MSC-818) on THP-1-derived macrophages treated with pro-inflammatory molecules/cytokines to mimic OA (1 µg/mL LPS, 10 ng/mL TNFα or 10 ng/mL IL1β) (Fig. 3a). We found that MSC-818 treatment of LPS-stimulated macrophages supressed *IL1B* (FC: 0.1; *P* ≤ 0.01), *IL6* (FC: 0.1; *P* ≤ 0.000 1) and *TNFA* (FC: 0.5; *P* ≤ 0.01) gene expression, with a trend for reduced expression of *NFKB*. Under TNFα stimulation, treatment with MSC-818 suppressed expression of *IL1B* (FC:0.2; *P* ≤ 0.000 1), *TNFA* (FC: 0.7; *P* ≤ 0.05) and *NFKB* (FC: 0.7; *P* ≤ 0.05). MSC-818 treatment also suppressed IL1β-induced *IL1B* gene expression (FC: 0.6, *P* ≤ 0.05) with a trend for lower *IL6* expression *(P* = 0.18). ELISA analysis of IL1β cytokine production by macrophages revealed that MSC-818 treatment suppressed IL1β concentration in the supernatant (FC: 0.7; *P* ≤ 0.05) following LPS stimulation (Fig. 3b). Collectively, these data indicate that exposure to CDK8i promotes an anti-inflammatory phenotype in THP-1-derived macrophages, which may translate to a reduction in inflammatory processes within the OA joint. Interestingly, modification of primary murine osteoclast function is also observed with CDK8i (Fig. S9), thus showing broad effect on cells from the macrophage lineage.

CDK8 has been linked to promoting glycolysis in tumour microenvironments,^28^ a process that is also upregulated in growth plate hypertrophic chondrocytes and at the articular surface in OA.^3–5^ We therefore sought to determine whether CDK8i exposure modulates mitochondrial function and if it is also beneficial as a modifier of the metabolic profile in OA chondrocytes. ATDC5 micromasses pretreated with IL1β or under chondrogenic or hypertrophy/mineralisation conditions were exposed to MSC-818 and then subjected to the MitoStress Test via the Agilent Seahorse system. This analysis showed that MSC-818 increased cellular ATP production rate under chondrogenic (100 nmol/L, FC: 1.4, *P* ≤ 0.000 1*;* 1 μmol/L, FC: 1.4; *P* ≤ 0.01) and hypertrophy/mineralisation conditions (100 nmol/L, FC: 1.5, *P* ≤ 0.001*;* 1 μmol/L, FC: 2.1, *P* ≤ 0.000 1) *(*Fig. 3c). Increases in glycoATP (via glycolysis) and mitoATP (via oxidative phosphorylation; OXPHOS) were equal under chondrogenic conditions, resulting in identical proportions of ATP generated from glycolysis across treatments. Under hypertrophy/mineralisation conditions, MSC-818-treated ATDC5 cells displayed a reduced reliance on glycolysis for ATP production, as supported by the dose dependent decrease in percentage ATP from glycolysis (100 nmol/L, FC: 0.8, *P* ≤ 0.001, 1 μmol/L, FC: 0.7, *P* ≤ 0.000 1). Moreover, it is evident that hypertrophy/mineralisation conditions reduced OXPHOS in ATDC5 micromasses, whilst MSC-818 dose-dependently protected from this loss of OXPHOS.

To identify proteins modulated by CDK8i under chondrogenic and OA-like conditions, Phospho-kinase array analysis was performed on ihMSCs differentiated towards chondrocytes and exposed to IL1β and/or MSC-818. This array is a multiplex immunoassay (a mini-Western blot on a chip), used here to rapidly screen the phosphorylation status of multiple kinase targets simultaneously in one sample. We found that both IL1β and MSC-818 treatment had a widespread effect on phosphorylation events (Fig. 3d). Proteins that were affected by IL1β in one direction and modulated by MSC-818 in the opposing direction were of particular interest as this would be consistent with an augmentation of OA-like targets by CDK8i. One such protein was HSP60, which was downregulated following IL1β treatment. Interestingly, a robust increase of HSP60 was observed after MSC-818 treatment under chondrogenic conditions, which was maintained upon dual IL1β/MSC-818 exposure. It is also interesting that HSP27 was modestly increased by MSC-818 and further increased by treatment in the presence of IL1β. Notably, STAT1 phosphorylation (a known CDK8 target), was downregulated by MSC-818 across both conditions, validating the activity of the compound within this disease model. As these data reveal that CDK8i induces a shift in the immune-metabolic response under hypertrophy/OA-like (IL1β) conditions, additional investigations on reactive species were conducted using reactive oxygen assays and nitric oxide colorimetric assays conducted on ATDC5 micromassess in the presence of MSC-818 and IL1β. These studies highlighted the modulation of both intra- and extracellular reactive oxygen and nitrogen species (ROS/RNS) by CDK8i (Fig. S10). Intriguingly, CDK8 expression and localisation are modified in chondrogenic conditions (vs IL1β OA-like; Fig. 3e). Indeed, through immunocytochemical analysis, IL1β stimulation increased CDK8 expression (13%; *P* ≤ 0.001) localised predominantly to cytoplasmic and perinuclear regions with punctate-like nuclear staining. This pattern was modified under chondrogenic conditions and may infer a cytoplasmic CDK8 function (i.e. mitochondrial fitness).

### CDK8i reduces disease markers, limits proteoglycan loss and corrects gait asymmetry in the STR/Ort spontaneous mouse model of OA

**Fig. 4:**
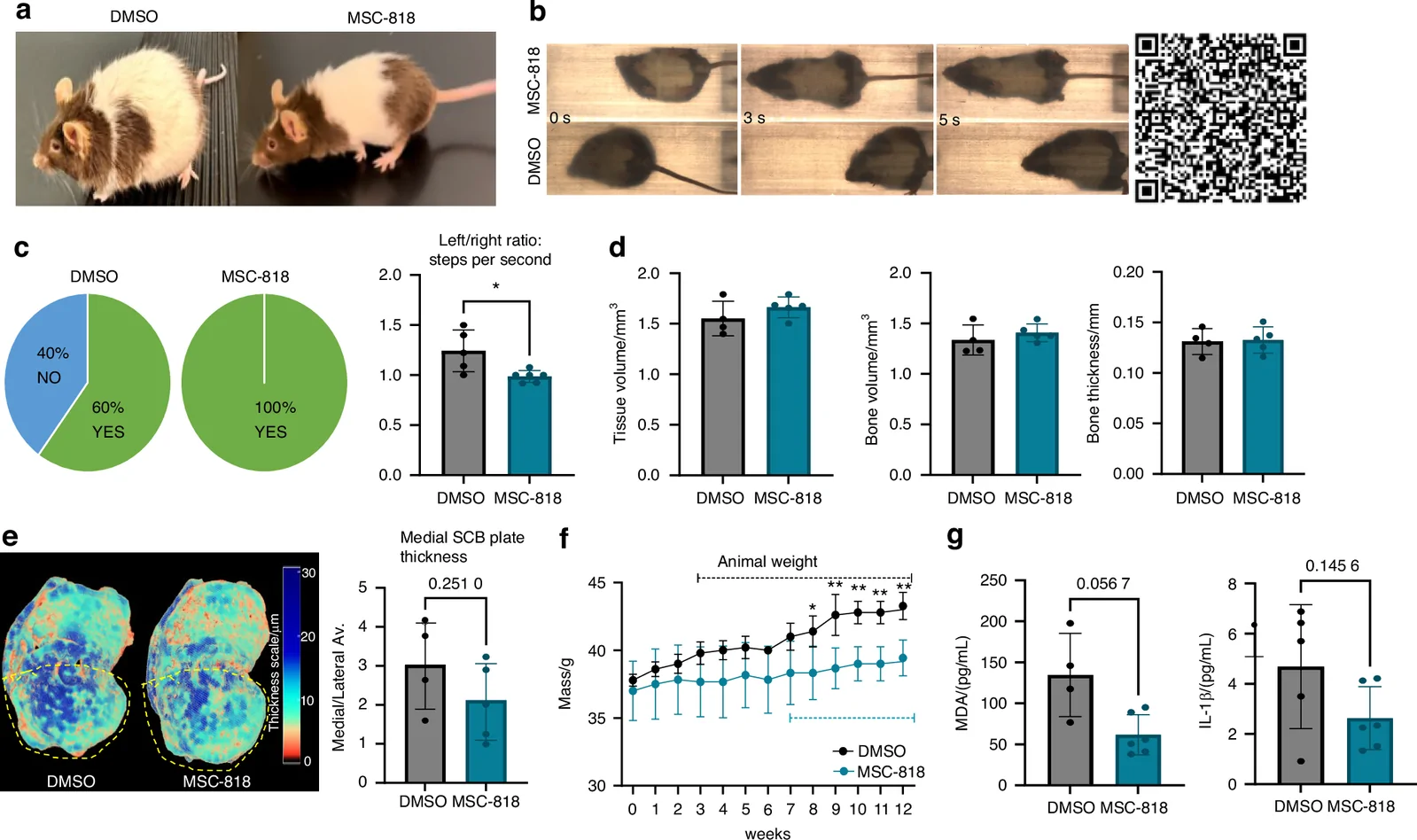
CDK8i treatment of the STR/Ort mouse model of aberrant chondrocyte hypertrophy/OA-like disease. **a** Appearance of DMSO versus MSC-818 treated STR/Ort mice. **b** Treadmill timelapse images of a STR/Ort mouse treated with MSC-818 (top) compared to DMSO control (bottom). QR code provides video link. **c** Compliance to running (pie charts) and gait symmetry assessment from treadmill analysis showing increased compliance to running and normalised step per leg ratio. **d** µCT analysis of right limb subchondral bone plate. **e** Subchondral bone plate thickness analysis of right limb medial tibial condyle. Representative CTVox pseudo-colour images with region of interest highlighted in yellow. Bone thickness (µm) is represented by the colour scale. (DMSO *n* = 4, MSC-818 *n* = 5), statistical analysis = Welch’s *t*-test, *P* = 0.25). **f** STR/Ort weight gains over the course of the study. Note significance in mass increase from week 0 began at week 3-12 of the study in control (DMSO) animals (black dotted line = *P ≤ 0.05). 5 mg/kg MSC-818 treated mice gained significantly less weight, with significance from the start of the study only evident at week 7-12 (teal dotted line = *P ≤ 0.05). Statistical significance between weekly weights = Šídák’s 2-way ANOVA, statistical difference between % weight increase = Mann Whitney Test. DMSO *n* = 5, MSC-818 *n* = 6, *P ≤ 0.05, **P ≤ 0.01, error bars = SD. **g** ELISA analysis of serum MDA (DMSO *n* = 4, MSC-818 *n* = 6) and IL1β (DMSO *n* = 5, MSC-818 *n* = 6). Statistical analysis = *t*-test with Welch correction

The male STR/Ort mouse exhibits spontaneous OA, closely resembling that observed in primary OA in the human population (articular cartilage degeneration, osteophytes, heterotopic ossification, synovial hyperplasia and subchondral bone sclerosis). These pathological histological features develop from 18-weeks with the full spectrum of OA pathologies evident at 35–40 weeks of age.^29–31^ Following 12-weeks of oral dosing (from 22 weeks old) with CDK8i (5 mg/kg MSC-818), STR/Ort mice (34 weeks old) appeared alert and active in addition to being well groomed, whilst untreated control mice were less mobile, larger in size and often piloerect (Fig. 4a). Gait analysis evaluated by treadmill task performed on week 11, when mice were 33 weeks of age, showed all MSC-818-treated mice were able to successfully complete the treadmill task with no contact with the bumper (Fig. 4b, c). In contrast, only 60% of the DMSO control group were able to complete the task. A step count ratio was calculated from the number of steps per leg during 5 s of active walking, which showed DMSO-treated mice to favour walking on their left leg over their right, indicative of gait asymmetry and possible right hind limb pain. Such asymmetry was not observed in the MSC-818-treated mice whereby a ratio of 1:1 was found across all mice, suggestive of equal gait. The right limb was therefore selected for further analyses.

It is well known that the tibia, and prominently it’s medial plateau, is the most OA-affected compartment of STR/Ort mouse knees.^32^ µCT analysis of the entire tibial subchondral bone plate showed MSC-818 to have no effect on bone parameters (bone volume, tissue volume and bone thickness; Fig. 4d). Surprisingly, in vitro analyses of bone cells found CDK8i to supress osteoblast mineralisation and osteoclast resorption (Fig. S9), suggesting that the observed lack of subchondral bone plate changes in response to CDK8i in vivo may involve the suppression of both cell types. Visual interrogation and 3D subchondral bone plate thickness showed the medial compartment to be thicker than the lateral, indicative of subchondral bone plate thickening across all animals. Following image analysis of the percentage of thickened area (defined by the area of dark blue from the 3D CTVox colour mapping, representing ~20–30 µm; Fig. 4e), the average lateral percentage area for the DMSO control group was 11.07% ( ± 4.02) whilst the MSC-818 percentage area was 8.66% ( ± 3.04), with a pooled average of 9.73% ( ± 3.50). This pooled average baseline thickness was used to generate a ratio of the medial “diseased” to “healthy” thickness (Fig. 4e). These analyses indicated that MSC-818 modifies subchondral bone parameters in areas of more progressed disease, with a trend towards reduced thickness ratio observed (FC: 0.7; *P* = 0.25), which aligns with reduced osteoblast and osteoclast activity in response to CDK8i.

During weekly weighing it was clear that control STR/Ort mice progressively gained weight in line with the literature.^30,33^ As shown in Fig. 4f, weekly reported weights of control mice displayed a 13.8% increase by week 12 compared to week 0, which was 2.3-fold lower in MSC-818 treated mice (FC: 0.4; *P* ≤ 0.01). Other studies have reported a weight range of 30–35 g for CBA mice, thus CDK8i treatment appeared to normalise body mass to that of the parental strain from the age of dosing.^34^ Analysis of known circulating OA biomarkers were analysed by ELISA to determine whether systemic MSC-818 also modulates inflammation and oxidative stress in STR/Ort mice. As shown in Fig. 4g, 5 mg/kg MSC-818 treatment led to lowering of both serum malondialdehyde (MDA; FC: 0.5*; P* = 0.056 7) and IL1β (*P* = 0.145 6), implicating the modulation of immunometabolism in the STR/Ort mouse. This also aligns with immunometabolic effects of in vitro MSC-818 treatment.

**Fig. 5:**
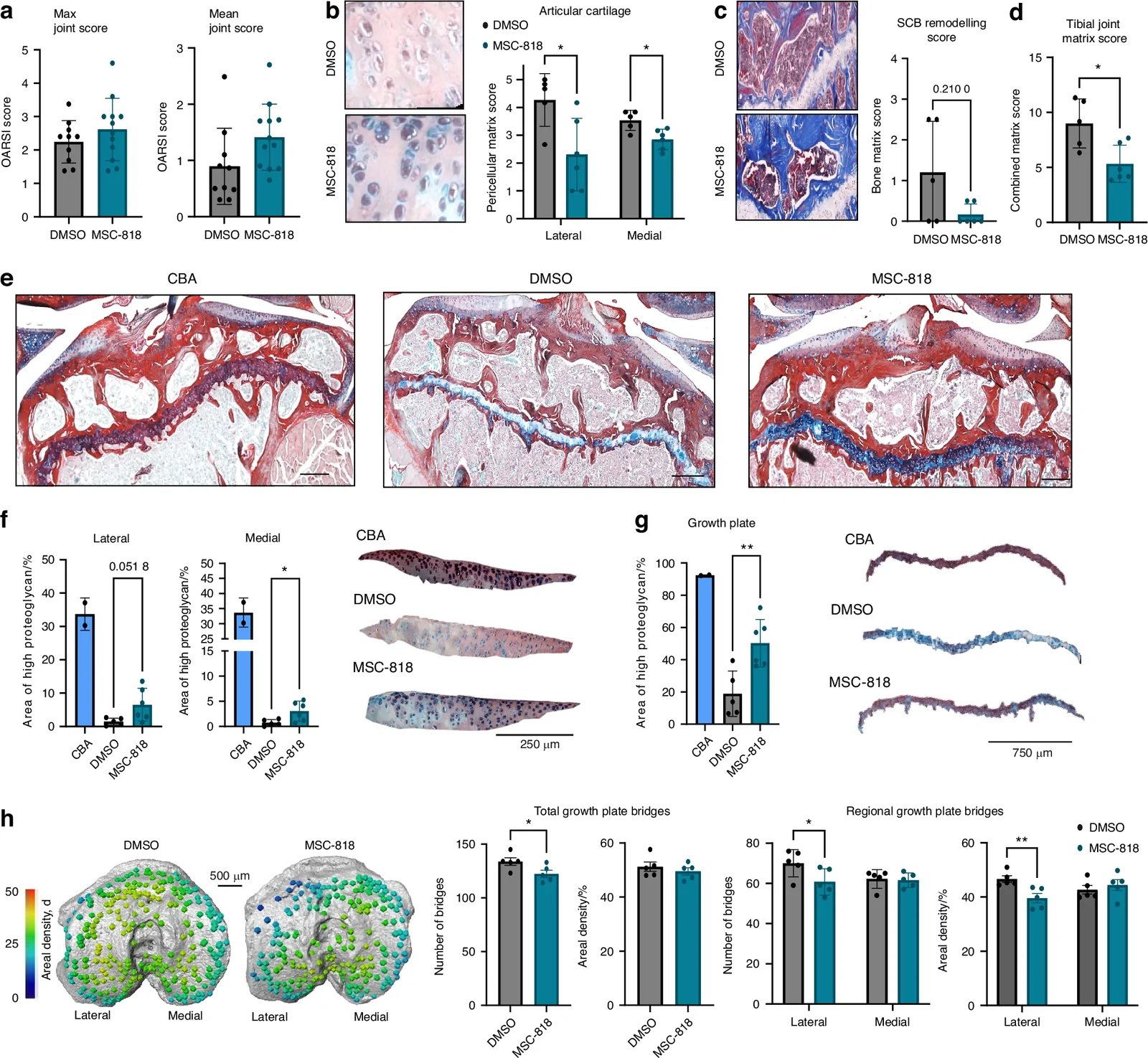
Joint matrix scoring and high proteoglycan quantification of CDK8i treated STR/Ort mice. **a** Left and right limb, mean and max joint OARSI scoring (DMSO; *n* = 10, CDK8i; *n* = 12). **b** Right hind limb pericellular matrix score of tibial (medial and lateral) articular cartilage with representative images (DMSO *n* = 5; MSC-818 *n* = 6). **c** Right hind limb bone matrix, subchondral bone remodelling score with representative images (DMSO *n* = 5; MSC-818 *n* = 6). **d** Combined pericellular and bone matrix score providing a complete tibial joint matrix score (DMSO *n* = 5; MSC-818 *n* = 6). **e** Representative images of RGB stained knee sections (Scale bar 250 µm). **f** Area of high proteoglycan quantification in the lateral and medial tibial articular cartilage, and **g** Growth plate of vehicle (*n* = 5) and CDK8i (*n* = 6) STR/Ort and parental CBA control strain (*n* = 2) with representative images; (**a**–**g** average score of 3 individual sections per mouse from 2 independent scorers; Error bars = SD). **h** Total and regional tibial growth plate bridge number and density, representative image with bridge location and areal density mapped in colour. Error bars = SEM (DMSO *n* = 5; MSC-818 *n* = 5). Statistical analysis a-d; Mann Whitney, (**e**, **f**); Welch’s *t*-test, (**g**); Two-way ANOVA *P ≤ 0.05, **P ≤ 0.01

Despite the observed shifts in serum parameters, gait, weight and overall appearance, OARSI scoring of Toluidine blue stained histological sections from the right knee revealed no differences in OA lesion score between vehicle and MSC-818-treated STR/Ort mice (Fig. 5a and Fig. S11). This may be due to the treatment commencing at a post-OA phase in this model, but nonetheless this OARSI scoring failed to align with the functional improvement in limb use observed in MSC-818-treated mice. As such, we sought to devise a histological scoring system that could complement OARSI scoring via the detection of distinct parameters in the chondrocyte matrix and cellular responses to treatment which may instead be linked with tissue regeneration (Fig. S12). It was deemed that bone matrix analyses should also be included to allow interrogation of effects on early remodelling linked to bone sclerosis/OA bone pathology (Fig. S13).

RGB staining (combining Picosirius Red, Fast Green and Alcian Blue^35^) of sections from both CBA parental strain (comparator) and DMSO treated STR/Ort mouse knee joints revealed that STR/Ort mice displayed almost complete ablation of the proteoglycan rich chondrocyte pericellular matrix in the articular surface compared to parental CBA mice (Fig. 5b,e,f). This allowed for the generation of a chondrocyte pericellular matrix scoring system, whereby a score of 1 was given to healthy, prominent pericellular matrix which was stained dark blue/purple as observed in CBA sections; whilst a score of 5 was given to sections lacking pericellular matrix staining, commonly observed in the STR/Ort mouse sections. Scores 2–4 were allocated based on the pericellular matrix staining gradients observed in the STR/Ort mouse sections. An identical approach was adopted with the bone matrix scoring, with high proportions of new bone matrix given a score of 5 whilst little to no new bone matrix was given a score of 1, and higher scores (2–4) were allocated based on the proportion of new bone matrix.

Following RGB-based matrix scoring of both DMSO- and MSC-818-treated STR/Ort mice, it was evident that treatment with MSC-818 enhanced proteoglycan content across the lateral (FC: 4.6; *P* ≤ 0.05) and medial (FC: 4.1; *P* ≤ 0.05) tibial plateaus, resulting in a lower pericellular matrix score than in DMSO-treated STR/Ort mice. Similarly, MSC-818 limited new bone matrix formation, reducing the subchondral remodelling score (vs. DMSO, Fig. 5c). Combining these scores disclosed a lowering of the overall tibial matrix score by MSC-818 treatment in STR/Ort mice (FC: 0.6; *P* ≤ 0.05, Fig. 5d). Perhaps the most striking observation was in the growth plate matrix (Fig. 5g), wherein STR/Ort mice treated with DMSO displayed a global loss of proteoglycan (vs. parental CBA mice) that was rescued by MSC-818 treatment (FC: 2.6 vs. DMSO control). To link these changes in growth plate biology to those observed at the articular surface, we explored whether MSC-818 treatment also modified growth plate bridge formation, which has already been linked to OA predisposition in STR/Ort mice. Growth plate bridges are trans-physeal mineralised struts that traverse the bone-cartilage-bone growth plate interface, enabling the perpendicular connection of epiphyseal and metaphyseal bone surfaces.^22^ Although the precise function of these bridges is unclear, we have found that STR/Ort mice display more overt bridge formation and distinct spatial bridge clustering compared to the parental CBA line. It is postulated that aberrant bridging modifies joint mechanics, leading to cartilage degeneration and other hallmarks of OA. Indeed, increased number of growth plate bridges appear in more severely affected OA joints. Growth plate bridge analysis was therefore performed on repositioned µCT datasets in 3D, which disclosed that MSC-818 promoted reductions in bridge number (−7.97%, *P* ≤ 0.05) and areal density (−4.6%, *P* ≤ 0.01). Upon further inspection this involved a regionalised deficit exclusively within the lateral side (bridge number; −33.4% and areal density; −10.9%, vs. DMSO; Fig. 5h). These coincide precisely with the areas exhibiting greatest change in pericellular proteoglycan staining levels.

**Fig. 6:**
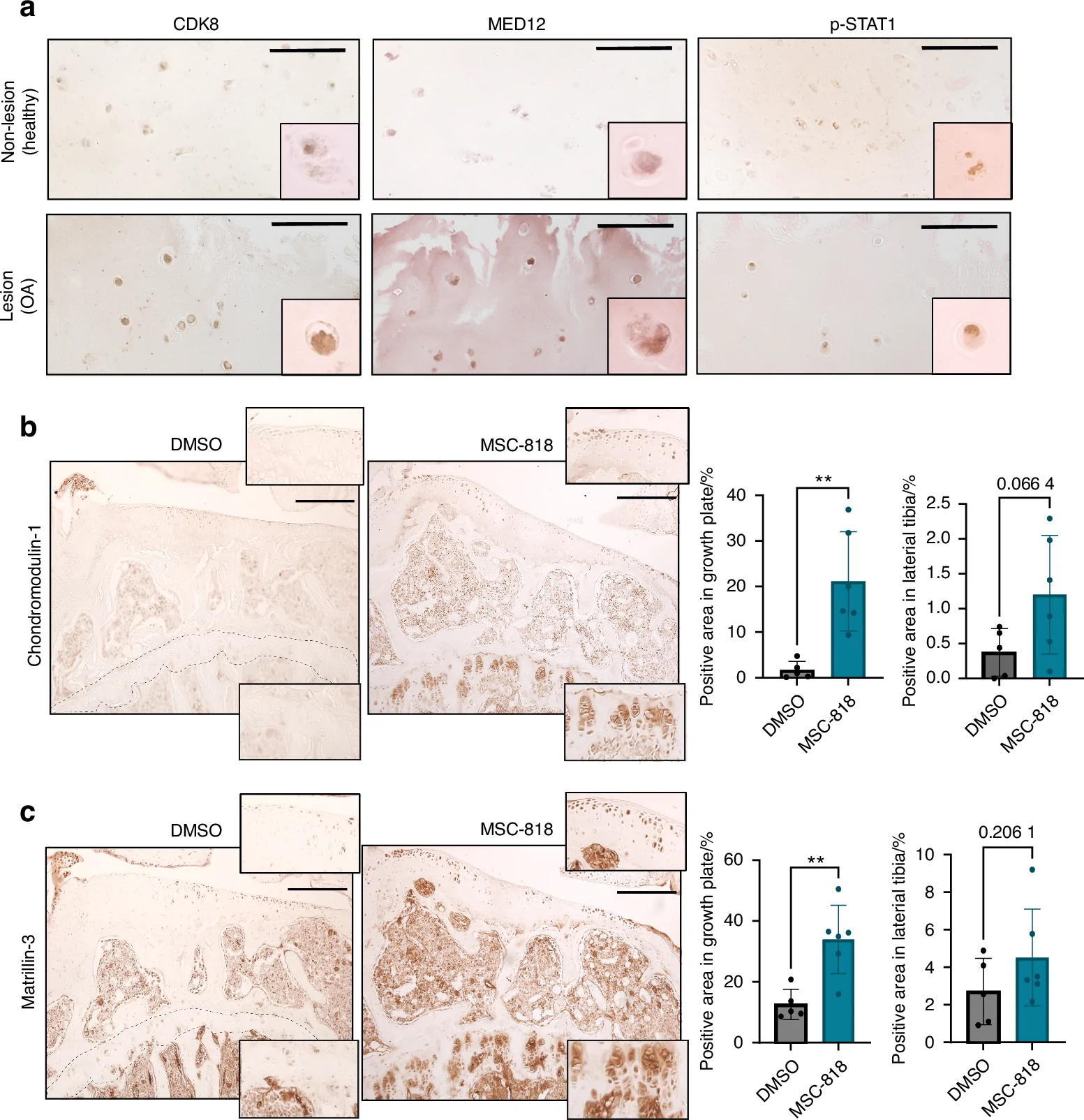
CDK8, MED12 and p-STAT1 expression/localisation in human cartilage and CDK8i biomarker expression in CDK8i treated STR/Ort mice. **a** CDK8, MED12 and p-STAT1 in human non-lesion cartilage (Mankin 5 ≥ 7) and OA-lesion cartilage (Mankin 8 ≥ 10) (Scale bar 125 µm; inset diameter 37.5 µm). **b**, **c** Chondromodulin-1 and Matrillin-3 (CDK8i biomarkers) in the growth plate and lateral tibial articular cartilage of MSC-818 or DMSO treated STR/Ort mice. Dotted line on DMSO images indicate the growth plate border (1 central knee section quantified per mouse; DMSO *n* = 5, MSC-818 *n* = 6), scale bar = 250 µm, error bars = SD, statistical analysis Welch’s *t*-test. **P ≤ 0.01

To further validate CDK8 as a therapeutic OA target, we mapped CDK8, its mediator complex co-factor MED12, and its known downstream target p-STAT1 (by immunolabeling^36^) in human non-lesion and OA-lesion cartilage (Fig. 6a). In non-lesion cartilage, CDK8, MED12 and p-STAT1 expression was limited to the nucleus and primarily localised to the deep zones of articular cartilage. In contrast CDK8, MED12 and p-STAT1 labelling was most prominent within chondrocytes (larger and likely hypertrophic) adjacent to lesions within the superficial zone in OA cartilage, where expression was mostly observed throughout the cell. This is consistent with an increased CDK8 expression seen in response to IL1β (Fig. 3e). Immunohistochemical analysis of CDK8i biomarkers [chondromodulin-1 and matrillin-3 identified in previous RNAseq studies (Fig. S5–6)] in STR/Ort mouse knee sections found MSC-818 to upregulate both biomarkers in the growth plate (chondromodulin-1; FC: 13.5; *P* ≤ 0.01, matrillin-3; FC: 2.7; *P* = 0.07) (Fig. 6b, c) and induce trends for greater expression in lateral tibial articular cartilage (chondromodulin-1; FC: 3.2; *P* = 0.07 and matrillin-3; FC: 1.7; *P* = 0.21), thus validating in vitro gene expression data.

## Discussion

**Fig. 7:**
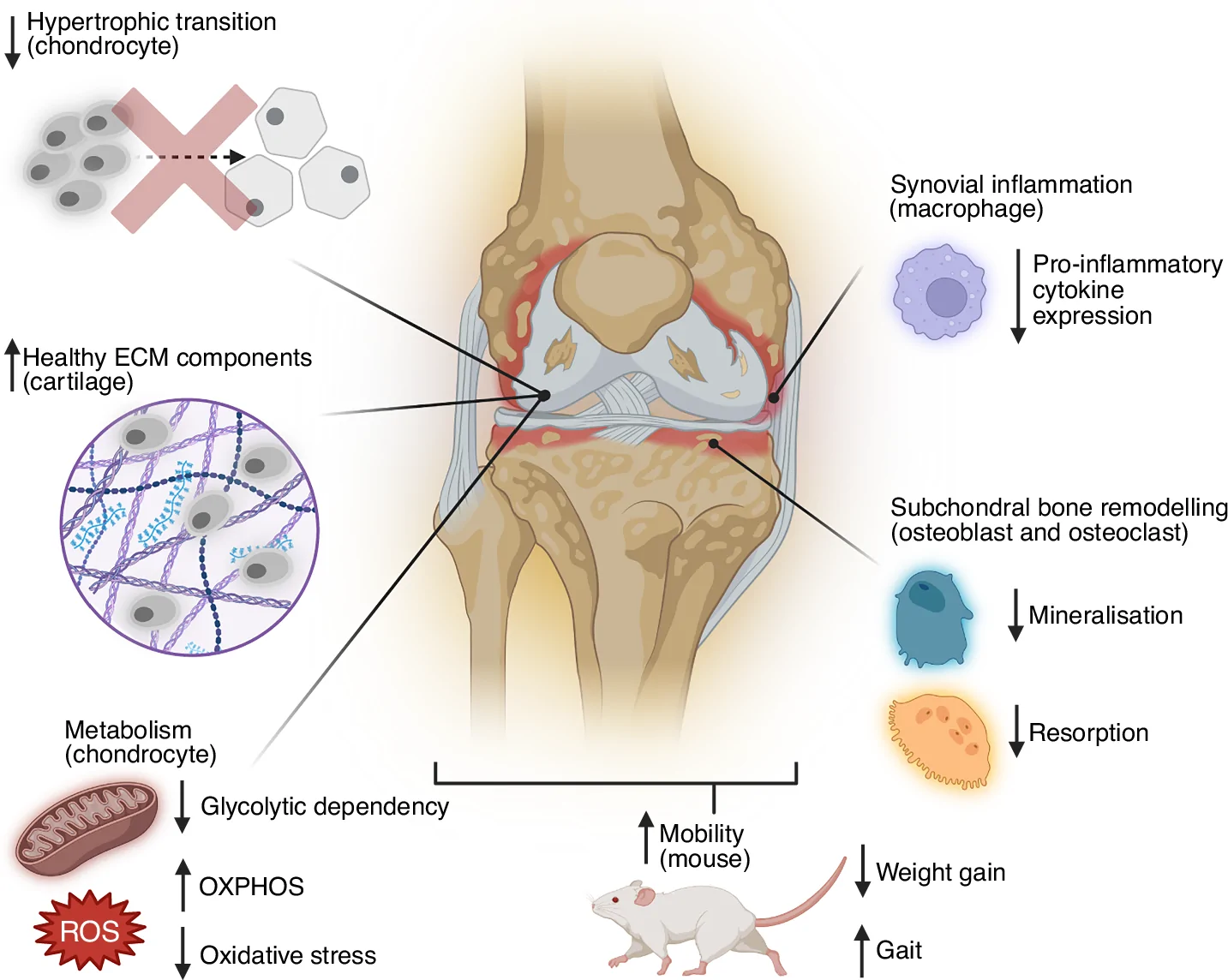
CDK8i has the potential to modify whole joint pathobiology in OA. From the data presented, CDK8i displays a pro-anabolic, anti-hypertrophic and anti-catabolic phenotype in chondrocytes (ATDC5, ihMSC and primary equine) with treatment restoring healthy metabolic processes. CDK8i suppresses pro-inflammatory processes in THP-1-derived macrophages, in addition to modulation of subchondral bone remodelling via suppression of primary murine osteoclast resorption and osteoblast (MC3T3) driven matrix mineralisation. In vivo, CDK8i promotes mouse mobility, suppresses weight gain and improves gait parameters whilst modulating biomarkers, subchondral bone parameters, chondrocyte pericellular matrix and bone matrix dynamics in the STR/Ort mouse spontaneous model of OA. Created in BioRender. Roberts, S. (2026) https://BioRender.com/ojm6og9

Through a screening campaign of a focused small molecule compound library containing modulators of transcriptional/epigenetic machinery, we identified CDK8 inhibition as a modulator of chondrocyte biology with relevance to OA via an association with endochondral ossification. CDK8i treatment was found to modify chondrocyte phenotype, resulting in pro-anabolic, anti-catabolic/-hypertrophic effects that translated across species and cell populations. CDK8i also increased OXPHOS capacity and suppressed the glycolytic dependency of in vitro chondrogenic cultures under hypertrophic conditions, likely promoting mitochondrial fitness via upregulation of HSP27/60.^37^ CDK8i exerted therapeutic effects beyond chondrocytes, extending to suppression of osteoclastic-mediated resorption and osteoblast-driven matrix mineralisation, with implied benefit of CDK8i in both early and late-stage OA subchondral bone pathology. Moreover, CDK8i displayed anti-inflammatory effects in LPS-, TNFα- and IL1β-stimulated macrophages that align with prior findings which showed that CDK8 promotes inflammation, whilst its inhibition suppresses inflammatory, and promotes anti-inflammatory processes across various cell types.^38–43^ Collectively, these in vitro data suggest that CDK8 inhibition modifies cartilage endochondral ossification and has potential whole joint disease-modifying efficacy in OA. Translation was evident in the OA modifications in STR/Ort mice seen upon in vivo CDK8i treatment, with reduction in serum OA biomarkers (IL1β and MDA), improved locomotion, suppressed pathological weight gain, modified subchondral bone thickness, fewer growth plate bridges, less local bone remodelling and increased expression of CDK8i biomarkers. These data highlight the potential of CDK8 inhibitors for whole joint disease modification in OA (Fig. 7).

CDK8 functions predominantly in transcriptional control via interactions with RNA polymerase II (RNA pol II) and Mediator Complex to exert gene activator and repressor roles.^44^ CDK8 phosphorylates a plethora of proteins (e.g. transcription factors such as STAT1, chromatin and epigenetic regulators, and the transcription machinery) to activate targeted gene expression to drive specific signalling pathways.^45,46^ As CDK8 shares 91% sequence homology and significant overlap in both kinase and cyclin domains with CDK19, current inhibitors of CDK8 function as dual CDK8/19 inhibitors.^47,48^ Whilst the kinase activity of CDK19 is undefined, it likely has different interactions to CDK8 due to variations in c-terminal kinase tail sequences,^49^ functioning instead as a scaffold protein to drive kinase-independent signalling, and serving to stabilise CDK8 for kinase-dependent processes.^50–52^ In mammals, the Mediator Complex is a large multi-subunit complex which is proposed to function as a bridge between transcription factors and RNA pol II, which includes a 600 kD kinase module consisting of Mediator Complex subunits 12 (MED12 or MED12L), 13 (MED13 or MED13L), CDK8/19 protein kinases and cyclin-C.^53,54^ Vitally, Mediator-driven CDK8 activation is predominantly actioned by MED12 association with cyclin-C, with cyclin-C destruction inactivating CDK8 during cell stress response.

Little is known about the role of CDK8 in chondrocyte or cartilage biology; however, much can be inferred from the literature. Indeed, CDK8 is known to regulate many core chondrocyte signalling pathways, including Wnt, TGFβ, BMP and T3-thyroid signalling. This presents the intriguing possibility that CDK8 is integral to chondrocyte phenotype and disease progression, although this remains to be tested experimentally. Interestingly, support for multifaceted roles of CDK8 in chondrocyte biology has arisen in a recent single-cell RNAseq study by Kodama et al. on the murine growth plate that found CDK8 was upregulated specifically in the resting zone, with re-expression observed in the hypertrophic zone.^55^ Further evidence for a connection between CDK8 and endochondral ossification is found through analysis of individuals carrying de novo CDK8 mutations, who display low birth length and short stature in addition to various other developmental defects.^56^ This is important as factors that suppress endochondral ossification, a process driving bone length, have been proposed as therapeutic targets for OA due to inhibition of chondrocyte hypertrophy.^11^ Importantly, the data we present herein collectively show that CDK8 inhibition functions to modulate the transition to the hypertrophic chondrocyte phenotype. Interestingly, CDK8 is recognised as a ‘common barrier’ in multiple cell systems, with CDK8i enhancing both fibroblast plasticity and pluripotent stem cell reprogramming.^57–59^ CDK8i has also been shown to stabilise naïve human pluripotent stem cell phenotype in vitro by preserving their epigenetic profile, while preventing aberrant DNA methylation events that serve to compromise their differentiation potential.^58^ Moreover, CDK8i treatment of acute myeloid leukaemia cell lines induces cell reprogramming via potent upregulation of super-enhancer-associated genes involved in controlling differentiation.^60^ These include MYC-dependent genes that block hypertrophic differentiation and stabilise the chondrogenic state.^61^ Likewise, our phased dosing studies show CDK8i induces robust phenotypic modification, with 48-h exposure exerting long-term stabilisation of chondrocyte phenotype, equivalent to CDK8i being present for the entire culture period.

Male STR/Ort mice develop OA spontaneously, which increases in severity with age.^30^ This model also exhibits an accelerated growth phenotype, with abnormal growth plate chondrocyte hypertrophy and a higher number and density of growth plate bridges, both of which correlate with OA progression.^22,62^ We find that CDK8i treatment of these mice for 12-weeks promotes a dramatic improvement in body condition and movement, despite having no significant effect on OARSI score. This may indicate either systemic modification in metabolic processes, modification of inflammatory/pain responses or the insufficiency of OARSI scoring to reveal therapeutically relevant cellular responses to treatment; likely a combination of all three. As such, we sought to develop a complementary scoring system based on proteoglycan content as an effector of cartilage biomechanics and subchondral bone as a predictor of sclerosis, that could be used to complement OARSI scoring to allow the monitoring of ECM dynamics. Using this RGB-based matrix scoring system, we observed a significant improvement in joint tissue parameters in STR/Ort mice treated with CDK8i. Our data also found that CDK8i treatment reduced the number of growth plate bridges either by suppressing their formation or by promoting their removal. The reduction in number and areal density of bridges found in the lateral compartment corresponds to lateral articular cartilage chondrocytes, showing a greater reduction in pericellular matrix score in response to MSC-818 treatment. This may be due to the fact that OA is less progressed laterally compared to medially.^63^ The finding that bridge dynamics can be modified by CDK8i to potentially stabilise the endochondral growth defect in the STR/Ort model further supports use of CDK8i as an OA therapeutic.

Despite clear improved joint matrix features in this preclinical model, the role of CDK8 in bone homeostasis remains controversial. Amirhosseini et al. found that CDK8 inhibition by Senexin B impeded mouse osteoclastogenesis in vitro. This was not, however, observed herein where pre-osteoclast/osteoclast number was unaffected by 1 μmol/L BI-13471. This may be explained by the distinct study designs; for instance, Amirhosseini et al. treated with Senexin B immediately after seeding bone marrow monocytes, while in our study BI-1347 treatment commenced 24-h post seeding/differentiation. Thus, inhibiting CDK8 in monocytes prior to osteoclast lineage commitment may result in fewer committed cells to begin with, rather than CDK8i impeding osteoclastogenesis overall. Further studies found that Senexin B promoted mineralisation of mouse primary osteoblasts (but not differentiation) in vitro.^64^ This has been supported by Yamada et al who found that an alternative CDK8i (KY-065) also exerted bone anabolic effects.^65^ These data, however, also conflict with our findings showing that osteoblast mineralisation is ablated by MSC-818 in vitro, and that there is reduced new bone formation within the subchondral bone of MSC-818-treated STR/Ort mice in vivo. It is noteworthy that Senexin B does display promiscuity and inhibits kinases, including MAP4K2, which may underpin these divergent bone effects attributed to different CDK8i.^66^ Likewise, there may be a local specificity in the CDK8i response, which would be consistent with our observed greater irregular woven bone response below the periosteum.^39^

We find that CDK8, its Mediator-complex cofactor MED12 and its target pSTAT1 are upregulated near human OA cartilage lesions, and in ATDC5 micromasses (CDK8 only) after IL1β stimulation to infer a direct role for CDK8 in cartilage processes. Interestingly, in healthy human cartilage, CDK8, MED12 and pSTAT1 expression were found to be restricted to the nucleus, whereas expression was more diffuse throughout the hypertrophic-like chondrocytes in regions adjacent to OA lesions. This sub-cellular shift in localisation was replicated for CDK8 in ATDC5 micromasses, where IL1β treatment promoted a cytoplasm/pericellular distribution. CDK8 is, however, for the most part considered a nuclear kinase, even though recent studies find CDK8 to localise to mitochondria where it functions to promote fission via Drp1 phosphorylation.^67^ This is intriguing as mitochondrial fission is also found to be aberrant in OA due to enhanced Drp1 phosphorylation, making it tempting to speculate that relocation of CDK8 contributes to the mitochondrial/metabolic dysfunction observed in OA chondrocytes.^68,69^

Although the roles of CDK8 in metabolism are not fully explored, it is known to upregulate glucose uptake via GLUT1/3, resulting in overreliance on glycolysis for energy production, with CDK8i resulting in impaired glucose uptake and glycolysis in colorectal cancer cells.^28^ Short-term ablation of glycolysis with 2-Deoxy-d-glucose (2-DG) indeed alleviates catabolic OA chondrocyte processes.^70^ This supports our data showing that MSC-818 reduces glycolytic chondrogenic cell dependency by protecting OXPHOS and promoting mitochondrial-driven ATP production in hypertrophy/mineralisation conditions. Depletion in mitochondrial respiration (OXPHOS and ATP production) is indeed evident in OA, suggesting CDK8i may protect and preserve mitochondrial processes in chondrocytes.^71^ It should, however, be noted that despite the analysis of mitochondrial function/metabolic profile replicating the known bias towards glycolysis in hypertrophic chondrocytes/OA, these assays were carried out in normoxic conditions. This may have resulted in skewing of magnitude of CDK8i effect, and analysis under hypoxia may provide further insights.

Our data indicate a downregulation of HSP60 in ihMSC-derived chondrocytes by IL1β treatment but a robust upregulation by MSC-818 treatment, indicating HSP60 is correlated with chondrogenic phenotype stabilisation. HSP60, an intracellular chaperone, controls protein folding under cell stress and has many other roles depending on its post-translational modification.^72^ Localised primarily to the mitochondrial matrix, HSP60 has been shown to be directly involved in controlling ATP generation through glutaminolysis.^73^ Thus, HSP60 upregulation by MSC-818 may help to generate the enhanced ATP levels via OXPHOS observed in this study. Crucially, HSP60 overexpression in chondrocytes in vitro and in vivo not only rescued IL1β-induced mitochondrial impairment but also mice displayed significantly thicker cartilage, reduced synovial inflammation and osteophyte formation in addition to improved gait profiles.^74^ Further exploration of energy metabolism in MSC-818-treated chondrocytes is thus needed to fully resolve the complex role that CDK8 plays in energy metabolism and its influence on chondrocyte phenotype.

Limitations do however, exist in our study. For example, we selected a single 5 mg/kg oral dose, which may have restricted the level of efficacy observed. Furthermore, dosing of STR/Ort mice post-OA onset also likely restricted the efficacy on lesion severity we detected. Likewise, duration of dosing was also potentially too short for cartilage regeneration via the orally administered route. Indeed, recent work has shown that very early CDK8-IN-1 dosing has efficacy against lesion emergence in a surgically-induced OA model.^75^ Nonetheless, this efficacy was solely attributed to CDK8 roles in cellular senescence, a notion already supported by studies into MSC biology.^65^ Herein, generation of a complementary joint scoring system incorporating RGB and Masson’s Trichrome stain revealed novel biology in cellular responses to treatment, indicating early-stage articular cartilage regeneration, otherwise uncaptured by traditional OARSI scoring. Notably, this anabolic phenotype is amplified in the growth plate, where chondrocytes are considered inherently unstable, transient and hypertrophic, strengthening the notion that the mature, anabolic phenotype is stabilised by CDK8i, potentially correcting the hypertrophic chondrocyte characteristics known to be evident in STR/Ort joints. This is further supported by analysis of growth plate bridges, a structural phenomenon linked to OA in this STR/Ort mouse model, which show reduced numbers and density in response to MSC-818 treatment, indicating disease modification. Additionally, in this specific cohort of STR/Ort mice, we observed robust left/right rear limb asymmetry, with mice favouring left leg walking. Although individual STR/Ort mouse joints show variation in OA emergence and speed of progression, with instances of both unilateral and bilateral severe disease, our previous studies have not previously reported such robust left leg bias. We have however seen specific parameters suggesting this may be the case using discriminate analysis, where right rear leg swing time differs from left.^76^ Further in-depth gait analysis would be required to define this in a preclinical study such as presented herein. Finally, the inclusion of ATDC5 cells transfected with CDK8 would provide confirmatory data for the ihbMSC rescue experiments described in Fig. 2.

In conclusion, this study reveals CDK8 to be a key regulator of endochondral processes, with effects that extend to the regulation of responses to inflammatory mediators and the functional suppression of osteoblast and osteoclast matrix modification that are beneficial in limiting subchondral bone sclerosis. Mechanistically, this multi-cell-type effect is likely driven by cell type-specific signalling roles for CDK8, where in chondrocytes these are likely achieved via modification in Wnt/TGF pathways, along with the ability to resist inflammatory insult and maintain metabolic fitness. Our multi-species approach to validation of a proposed chondrocyte phenotypic reset allows us to speculate that a CDK8i OA therapeutic approach would be efficacious across species. Likewise, our analysis of multiple cell types that may contribute to OA pathology improved the likelihood that this has potential benefit in the entire OA joint, leading us to present a novel therapeutic with promise to be a disease modifying OA drug.

## Materials and methods

### ATDC5 micromass culture

ATDC5 chondroprogenitor cells (RRID:CVCL_3894; RIKEN) were cultured in Dulbecco’s Modified Eagle Medium (DMEM)-F12 (ThermoFisher, Massachusetts USA), 5% foetal bovine serum (FBS; Sigma-Aldrich, Missouri USA), 1% Antibiotic-Antimycotic (Ab/Am; ThermoFisher), 10 µg/mL human-transferrin (Sigma-Aldrich), 30 nmol/L sodium selenite (Sigma-Aldrich) under standard conditions (37 °C, 5% CO_2,_ humidified environment). Micromass cultures consisted of a 2 or 10 µL droplet of 2.7 × 10^7^ cells/mL in either a 96 or 24-well plate, respectively. Cells were seeded in growth media for 2-h before adding differentiation media (DMEM-F12, 5% FBS, 1% Ab/Am, 5 µg/mL human-transferrin, 1x insulin-transferrin-selenium premix (ITS-G; ThermoFisher) for 7 days, and a further 7 days in mineralisation media (alpha-MEM; ThermoFisher, 5% FBS, 1% Ab/Am, 7 mmol/L β-glycerophosphate; Sigma-Aldrich), 50 µg/mL L-ascorbic-acid-2-phosphate (Sigma-Aldrich), 5 µg/mL human-transferrin, 1x ITS) to induce chondrocyte hypertrophy/mineralisation.^77^

Micromasses were fixed in ice-cold 95% methanol for 25 min at −20 °C before staining with 0.5% w/v Alcian blue 8GX (ThermoFisher) in 1 mol/L HCl for 25 min at room temperature to visualise GAG deposition. Plates were imaged using an Epson Scanner (V370) at 1 200 dpi. To quantify, Alcian blue stain was leached from masses via incubation with 8 mol/L guanidine HCl (ThermoFisher) for 1–2-h at 37 °C with gentle agitation and absorbance measured at 650 nm using Spark®Cyto400 plate reader (Tecan, Zürich, Switzerland). To assess matrix mineralisation, micromasses were fixed in 4% paraformaldehyde (PFA; ThermoFisher) for 5 min at room temperature before staining with 1% w/v Alizarin red (ThermoFisher) for 5 min and washed with 50% EtOH. To quantify, Alizarin red was leached from masses via incubation with 10% cetylpyridinium chloride (CPC; Sigma-Aldrich) for 1-h at 37 °C with gentle agitation before absorbance being measured at 570 nm.

### Bulk RNAseq of BI-1347 (CDK8i) treated ATDC5 micromasses

ATDC5 cells were seeded in 10 µL micromasses (2.7 × 10^5^ cells) and differentiated in the presence of 1 μmol/L BI-1347 (CDK8i) or DMSO (*n* = 3/condition) for 7-days before RNA extraction using the ReliaPrep™ RNA extraction kit (Promega, Wisconsin USA). Total RNA quantification and integrity was determined via Bioanalyzer 2100 (Agilent, California USA). Samples with an RNA Integrity Number above 9.0 were processed for RNA sequencing (NovoGene, Cambridge UK) and aligned to the *Mus Musculus* reference genome (GRCm38/mm10), with differential expression, pathway enrichment and protein-protein interaction analysis conducted as previously described.^78^ Genes of interest relating to chondrocyte hypertrophy and cartilage homeostasis were identified.

### CDK8i treatment of ATDC5 micromasses

ATDC5 cells were seeded in 2 µL micromasses (5.4 × 10^4^ cells) and differentiated in the presence of different CDK8i representing distinct chemical series (MSC2530818; MSC-818 (BioOrbyt, Cambridge UK), BI-1347 (Boheringher, Ingleheim Germany; OpnME, APExBIO; Texas USA), BRD6989 (APExBIO), AS2863619 (APExBIO), CCT251545 (APExBIO) and SEL120-34A (Selleck Chemicals, Texas USA) at serial diluted concentrations ranging from 10 μmol/L–1 nmol/L or DMSO and processed for Alcian blue and Alizarin red staining on day 14.

For gene expression analysis, ATDC5 cells were cultured in 10 µL micromasses in the presence of BI-1347 or MSC-818 at 1 μmol/L, 100 nmol/L, 10 nmol/L or DMSO. On day 14, micromasses were processed for RT-qPCR. Briefly, RNA was extracted and converted to cDNA using the High-Capacity Reverse Transcription Kit (Applied Biosystems, Massachusetts USA) according to manufacturer’s instructions. qPCR was performed on each sample in duplicate using qPCRBIO SyGreen® Blue Mix Lo-ROX (PCR Biosystems, London UK) with data acquisition using Bio-Rad CFX96 (California USA). The expression of genes of interest was calculated relative to the average expression of the housekeeping genes (TBP and HPRT1) via the delta-Ct method.

To examine the longevity of the effect observed with CDK8i treatment, ATDC5 micromasses were exposed to a phased dosing regimen. Briefly, 10 µL ATDC5 micromasses were cultured in differentiation media containing 1 μmol/L of BI-1347 or MSC-818 for 48-h, before switching to control media containing DMSO for the remaining culture period. This was repeated for 4, 6, 9, 11 and 14 days of treatment. On day 14, cells were fixed and stained with Alizarin red or Alcian blue. MSC-818 was selected as the optimal compound going forward.

### Primary chondrocyte isolation and culture

Equine articular cartilage was harvested from the forelimb joints of skeletally mature horses (ages 3–24; *n* = 6), with ethical permission from the Royal Veterinary College Ethics and Welfare Committee (URN**-**2021-2064-2). All tissues were sourced from horses euthanised for reasons unrelated to this study, and other than joint injury. Isolated cartilage was placed into a petri dish containing phosphate buffer saline (PBS) supplemented with 100 µg/mL Primocin® (PBS-Ab/Am; Invitrogen, Massachusetts USA). Cartilage shavings were minced using a sterile scalpel to approximately 11 mm^3^ and washed in fresh PBS-Ab/Am. The cartilage pellet was resuspended in 5 mL TrypLE™ (ThermoFisher) prior to incubation at 37 °C for 10 min with gentle agitation. Dissociated cartilage fragments were pelleted and washed to remove excess TrypLE™ and resuspended in DMEM-complete media (DMEM/F12, 100 µg/mL Primocin®, 10% FBS) supplemented with 2 mg/mL Collagenase Type II (Sigma-Aldrich) and incubated overnight at 37 °C with gentle agitation. The cartilage digest was pelleted, washed and subsequently resuspended in DMEM-complete. Released cells were counted using a haematocytometer and seeded at 10 000 cells per cm^2^. The cultures were incubated under standard conditions for 2 days before refreshing DMEM-complete every 2–3 days until confluency.

### IL1β-induced model of OA ECM degeneration

IL1β is a potent inducer of cartilage degradation and it’s expression is significantly upregulated in the synovial fluid of patients with OA.^79^ To mimic this route of cartilage degradation in vitro, 10 µL ATDC5 micromasses were differentiated in the presence of 100 nmol/L MSC-818 for 12 days before induction of matrix degradation by co-treatment with IL1β supplemented medium (DMEM/F12, 5% FBS, 1% Ab/Am, 10 ng/mL human IL-1β; PeproTech, ThermoFisher) for 48-h.

Additional experiments were carried out on primary equine chondrocytes and ihMSC (TERT immortalised human mesenchymal progenitor cell line; ATCC SCRC-4000). Briefly, ihMSCs were seeded at a density of 5 × 10^4^ cells per 10 µL micromass and differentiated in ihMSC chondrogenic media (DMEM/F12, 1% FBS, 1%Ab/Am, 1x ITS, 10 μmol/L Y-27632 (APExBio), 100 nmol/L dexamethasone (Sigma-Aldrich), 50 µg/mL L-ascorbic-acid-2-phosphate, 20 ng/mL human TGF-β3; Peprotech). Primary chondrocytes were seeded at 2.5 × 10^5^ cells per 10 µL micromass and cultured in primary chondrocyte media [DMEM/F12; 2% FBS, 100 µg/mL Primocin®, 1x ITS, 10 ng/mL human TGFβ3 (PeproTech)]. All micromasses were differentiated for 12 days in the presence of 100 nmol/L MSC-818 or DMSO. Some micromasses were processed for gene expression analysis or GAG deposition via Alcian blue staining on day 12, whilst others were treated with IL-β media (DMEM/F12, 1% FBS, 1% Ab/Am, 10 ng/mL human IL-1β; PeproTech) with DMSO or 100 nmol/L MSC-818 for 48-h before processing on day 14 for Alcian blue staining/gene expression analysis.

### Generation of CDK8-overexpressing immortalised human bone marrow MSCs (ihbMSC)

The full-length wild-type CDK8 open reading frame was cloned between the XbaI and NotI restriction enzyme sites of lentiviral expression vector pCDF1-MSC2-EF1-Puro. Lentiviral particles were generated by transient transfection of HEK293T cells with a third-generation lentiviral system comprising the packaging plasmid psPAX2 (Addgene plasmid #12260), the envelope plasmid pMD2.G (Addgene plasmid #12259), and the pCDF1-MSC2-EF1-Puro vector encoding the CDK8 wild-type or mutant transgene under the control of the EF1α promoter. HEK293T cells (ATCC CRL-3216) were maintained in DMEM supplemented with 10% FBS (Gibco) at 37°C in a humidified incubator with 5% CO₂. For virus production, cells were seeded in 10 cm tissue culture dishes and transfected at 70%–80% confluence using Lipofectamine 2000 (ThermoFisher). Viral supernatants were harvested at 48-h and 72-h post-transfection, centrifuged at 500 × *g* for 10 min to remove debris, and passed through a 0.45 µm polyethersulfone (PES) filter (Millipore, Massachusetts USA).

Lentiviral supernatants were used to infect primary mesenchymal cells immortalised by expression of hTERT (Applied Biological Materials, Vancouver Canada). Cells were passaged in PriGrow II (Applied Biological Materials), 10% FBS and 10^−6 ^mol/L hydrocortisone in PriCoat™ Flasks (G299) or culture vessels coated with Applied Cell Extracellular Matrix (Applied Biological Materials). Stable pools expressing CDK8 were established by selection with 1 µg/mL puromycin (Sigma-Aldrich). All lentiviral work was performed in a biosafety level 2 (BSL-2) facility, in compliance with institutional biosafety regulations and under the approval of the local institutional biosafety committee.

Generated immortalised human bone marrow mesenchymal stem cells (ihbMSCs) were differentiated in micromasses under the same chondrogenic conditions as the ihMSCs, detailed above.

### ihMSC phospho-kinase array

ihMSCs were seeded into 10 µL micromasses and differentiated in the presence of DMSO or 100 nmol/L MSC-818 for 12 days for analysis under chondrogenic conditions. Some micromasses were further treated with IL1β differentiation media containing DMSO or 100 nmol/L MSC-818 for 48-h. On days 12 (-IL1β) or 14 (+IL1β), micromasses were washed with ice-cold PBS, and 24 micromasses were pooled per condition in lysis buffer (R&D Systems, Minnesota USA). Lysates were resuspended multiple times and incubated at 4 °C with gentle agitation for 30 min, before centrifugation at 14 000 × *g* for 5 min and quantified using Bradford Reagent (Sigma-Aldrich). Protein abundance and phosphorylation events were analysed using a human phospho-kinase array kit, according to manufacturer’s instructions (R&D Systems). Array blots were imaged using LiCor Odyssey FC. Pixel density for each spot was determined using the QuickSpots image analysis software, and the average pixel density was calculated before normalising to reference spot intensities.

### THP-1-derived macrophage generation/analysis

THP-1 monocytes (ATCC) were seeded at 1 × 10^5^ per cm^2^ and differentiated into macrophages via treatment with 50 ng/mL phorbol 12-myristate 13-acetate (PMA; SinoBiological, Beijing, China) in THP-1 growth media (RPMI-1640; ThermoFisher, 100 µg/mL Primocin®, 10% FBS, 0.05 mmol/L β-mercaptoethanol; Sigma-Aldrich) for 48-h. Wells were then washed to remove PMA, and media was replaced with THP-1 growth media for a 24-h rest period. Macrophages were then subjected to a 1-h pre-treatment with 100 nmol/L MSC-818. Inflammatory stimulation was then initiated through the addition of 1 µg/mL lipopolysaccharide (LPS; Sigma-Aldrich), 10 ng/mL human TNFα (Peprotech) or 10 ng/mL human IL1β (Peprotech) for 24-h in the presence of 100 nmol/L MSC-818. Gene expression analysis was conducted as previously described above. For LPS stimulated cells, media supernatant was used for IL1β cytokine quantification via ELISA (PeproTech, ABTS Human IL1β development ELISA) as per manufacturer’s instructions.

### MitoStress test

ATDC5 cells were seeded at 4 × 10^4^ in 1.5 µL micromasses in growth media and Seahorse XF Pro M Cell Culture Microplates for 2-h to allow cells to adhere, before addition of 200 µL ATDC5 differentiation media containing DMSO, 100 nmol/L MSC-818 or 1 μmol/L MSC-818. Cells were differentiated for 6 days before some wells were exposed to hypertrophy/mineralisation conditions (mineralisation media) or IL1β (ATDC5 IL1β media) for 48-h in the presence of DMSO or MSC-818 (100 nmol/L or 1 μmol/L). The cultures were conducted in normoxic conditions.

The XFe96 sensor cartridge (Agilent) and cells were prepared for the MitoStress Test according to manufacturer’s instructions using 0.5 μmol/L Rotenone (ApexBio), 0.5 μmol/L Antimycin A (Sigma-Aldrich), 1.5 μmol/L Oligomycin (APExBio) and 2.5 μmol/L BAM15 (Neuromics, Minnesota, USA). Analysis was carried out using Seahorse analytics integrated cloud-based analysis platform (Agilent).

### STR/Ort preclinical study

Male STR/Ort mice aged 22 weeks (post OA onset^30^) from an internal colony (Royal Veterinary College (RVC), London) were housed in polypropylene cages with environmental enrichment and a 12-h light/dark cycle at 21 °C ( ± 2 °C). Mice had access to RM1 standard diet (LBS Biotechnology) and water ad libitum. All procedures were approved by RVC ethics board and performed according to ARRIVE guidelines, in fulfilment of the UK Animals (Scientific Procedures) Act 1986. Each mouse was weighed daily throughout the study and dosed orally with either DMSO (*n* = 5) or 5 mg/kg MSC-818 (*n* = 6) dissolved in 60% PEG400 dH_2_O with a final DMSO concentration of 5%, 3 times a week intermittently for 12 weeks. Animals were then culled using Schedule 1 procedures.

#### Gait analysis

On week 11, mice (DMSO; *n* = 5, MSC-818; *n* = 6) were placed on a DigiGait™ (MouseSpecifics, Massachusetts USA), which is a treadmill-based video capture system that allows for belt speed selection. For data collection, each mouse was placed on the DigiGait™ and allowed to acclimatise for 5 min before the speed was set to 13 cm/s for 10 s with videos captured of mice whilst walking. Videos were then cropped to 5 s of active walking and number of steps per hind limb were manually counted for each mouse.^76^ Mice that could not walk freely away from the chamber bumper were classified as non-compliant; however, their step count was still included in the analysis.

#### Serum analysis

Blood was collected immediately post-mortem by cardiac puncture and terminal bleed. Samples were maintained at room temperature for 30 min before centrifugation at 4 °C (1 500 × *g* for 10 min). The top layer of serum was extracted and flash-frozen on dry ice before storage at −80 °C. Serum analysis of malondialdehyde (MDA; marker of oxidative stress; Abcam, serum diluted: 1:8) and IL1β (marker of inflammation; Abcam SimpliStep, serum dilution 1:5) was carried out in duplicate via ELISA as per manufacturer’s instructions.

#### Micro-CT and image analysis

Mice were sacrificed following 12-weeks of dosing as described above and hind limbs fixed in 4% PFA for 48-h before long term storage in 70% EtOH. X-ray micro computed tomography (µCT) was performed using the Skyscan 1172 (Bruker, Massachusetts, USA) on the right hind limbs to analyse MSC-818 effects on subchondral bone. Hindlimbs were wrapped in non-PVC plastic film before scanning to prevent drying and imaged at a 5 µm voxel size using X-ray tube energy of 50 kV and 200 µA with 960 ms exposure time with a 0.5 mm aluminium filter. Projection images were reconstructed into tomograms using NRecon 1.7.3.1 (Bruker) and repositioned using Dataviewer 1.5.4 (Bruker). Tibial subchondral bone plate regions were manually segmented and analysed using CTAn 1.18.4 (Bruker) and their thickness measured in 3D, using files from CTAn imported into CTvox 3.3.1 (Bruker) and a colour map applied to the segmented region. In line with the literature that suggests disease is more prominent in the medial condyle of STR/Ort mouse knees, it was apparent that the medial tibial subchondral bone was thicker than the lateral condyle potentially indicative of OA-related sclerosis.^30^ Image analysis in ImageJ (National Institutes of Health (NIH), Bethesda, USA)^80^ was used to compare the percentage area of the medial condyle that was sclerotic between the DMSO and MSC-818-treated STR/Ort. Briefly, the CTVox 3D pseudo-colour map image was converted to an 8-bit image before background removal and thresholding so that only dark blue regions (thicker) were analysed. The average lateral percentage area of thickened subchondral bone plate across all mice was determined as a baseline healthy thickened area measurement which was used to calculate a ratio of individual thickened medial condyle/average lateral condyle score for all mice.

#### Growth plate bridge analysis

Growth plate bridge analysis was performed on repositioned µCT datasets in 3D as described in Staines et al.^81^ Briefly, tibiae were segmented in Avizo 3D (V2022.2, Thermofisher) using the region-growing algorithm prior to manual selection of the central regions of individual growth plate bridges spanning the width of the growth plate across all slices viewed from multiple image planes (xy, xz and yz). Spatial distribution of growth plate bridges was determined using a bespoke in-house python script implemented in Avizo 3D (V2022.2) following the projection of growth plate bridges upon the tibial joint surface with pseudo-colours reflecting the areal density at the bridge location.

#### Histological analysis

Hindlimbs were decalcified in 10% EDTA solution at room temperature with gentle agitation for 7–10 days. Limbs were then blinded before being paraffin embedded in a sagittal orientation and 6 µm coronal sections cut throughout the entire joint with approximately 5 sections per slide. Every 4th slide across the entire joint was selected for Toluidine blue staining and subsequent OARSI scoring. Slides were dewaxed and rehydrated via a series of xylene/ethanol washes before incubation in acetate buffer at pH 5.6 (sodium acetate, 10% acetic acid) for 1 min. Slides were subsequently stained with Toluidine Blue solution (0.1% w/v in sodium acetate buffer pH 5.6) for 2 min before washing in dH_2_O and incubating in 100% acetone for 2 min. This step was then repeated in fresh acetone before a final incubation in xylene for 5 min. Sections were then mounted in DPX (Sigma-Aldrich).

Articular cartilage regions were scored according to the internationally recognised OARSI scoring system, which accounts for lesions only.^9^ Briefly, one section from each slide was graded using OARSI scoring criteria. Scoring was completed for all four condyles (medial/lateral, tibia/femur) on one section per slide by two independent blind scorers. The average score for each condyle of each joint was calculated for each scorer, which was then averaged to give an overall mean condyle score. The mean of combined condyle average scores was determined to establish the mean joint score to measure the extent of the articular cartilage damage. To determine OA lesion severity, the maximum score for each condyle and average of all condyle maximum scores were recorded. As STR/Ort mice develop OA asymmetrically, each limb was considered a biological replicate for OARSI scoring.

#### RGB stain for assessment of chondrocyte pericellular matrix

The novel RGB staining method developed by Gaytan et al. combines Picosirius red, Alcian blue and Fast green to distinguish chondrocyte pericellular/ECM, with dark purple indicating high proteoglycan content.^35^ Briefly, STR/Ort right knee sections (3 central sections per mouse, 4–5 slides apart) and 40-week male CBA (STR/Ort parental strain, comparator) were gradually rehydrated before incubation with 1% Alcian blue in 3% acetic acid for 20 min, then washed for 5 min in tap water. Sections were then stained with 0.04% Fast Green dissolved in dH_2_O and washed for 5 min in tap water, followed by staining with 0.1% Picosirius Red for 30 min. Sections were then washed twice for 5 min in 1% acetic acid before dehydration and mounting in DPX. All staining was carried out at room temperature. In line with the 3Rs principle, 40-week-old CBA mouse sections from historic samples were used as a comparator. Unlike the STR/Ort mouse, which shows a progressive increase in OA score between 20 and 40 weeks, this is not observed with CBA mice. Indeed, CBA mice are relatively protected from OA, thus making 40 weeks a suitable comparator for our study.^29,31^

#### Masson’s trichrome stain for assessment of bone matrix

Masson’s Trichrome is a well-established staining method that allows for distinction between mature (stained blue) and immature bone (stained red) matrix,^82^ revealing remodeling/modelling processes. Briefly, male STR/Ort right hind limb central knee sections (3 per mouse, 4–5 slides apart) and 40-week CBA comparators (1 central section per mouse) were gradually rehydrated before staining with Bouin’s solution (preheated to 58 °C) for 15 min. Sections were then washed with dH_2_O and stained with Modified Weigert’s Iron Haematoxylin for 5 min in the dark before differentiation in acid-alcohol solution for 15 s. Slides were then washed in dH_2_O and stained with Biebrich Scarlet-Acid Fuchsin solution for 5 min before re-washing and incubation in 1% Phosphomolybdic acid for 2 min. Slides were then directly incubated with 1.8% Aniline Blue for 5 min before washing in dH_2_O and then 1% acetic acid for 30 s. Slides were then washed with dH_2_O and gradually dehydrated before mounting in DPX and imaged.

#### Joint matrix scoring

A chondrocyte pericellular matrix scoring system was then devised to characterise cartilage matrix parameters (Fig. S12). Likewise, a bone matrix scoring system was used to assess subchondral bone remodelling (Fig. S13), a phenomenon that occurs in early/mid OA, which later results in subchondral bone sclerosis and osteophyte formation typically observed at advanced OA stages. To provide an overall tibial joint score that incorporates both cartilage and bone matrix, joint sections were independently scored by two blinded scorers. For both RGB (cartilage matrix) and Trichrome (bone matrix) stains the scores from the three slides were averaged, providing an average matrix score per mouse for each individual scorer. This score was then further averaged across the two scorers. If heterogeneity of matrix parameters was observed across the section, then individual regions were quantified, and an average score was calculated. Individual and combined (cartilage+bone) matrix scores were subsequently plotted. RGB-stained and imaged sections were further analysed to objectively quantify proteoglycan/collagen content. Briefly, lateral and medial tibial articular cartilage and the growth plates were individually isolated using ImageJ (NIH).^80^ Images were converted to 8-bit and threshold analysis was conducted at 110 (growth plate) or 120 (articular cartilage) pixel value, prior to the recording of the percentage area of high proteoglycan.

### Immunohistochemistry

Human cartilage sections were a kind gift from Prof. Gerjo van Osch (Erasmus MC, Rotterdam, Netherlands). Non-lesion (Mankin score 5–7) and OA lesion (8–10) cartilage sections were gradually rehydrated before overnight antigen retrieval with UNI-TRIEVE (Innovex Biosciences, Richmond, CA, USA) at 40 °C according to manufacturer’s instructions. Sections were then permeabilised using 0.1% Triton X-100, Tris-Buffered Saline (TBS) for 15 min at room temperature. Endogenous peroxidases were blocked using 0.3% hydrogen peroxide at 37 °C for 15 min prior to three 5-min washes in 0.1% TBS-Tween20. Sections were incubated in block buffer (10% goat serum, 0.1% bovine serum albumin TBS; Sigma-Aldrich) for 1-h (room temperature) before washing three times for 10 min in 0.1% TBS-Tween20, followed by overnight incubation at 4 °C with anti-CDK8 (Invitrogen PA5-115001; 1:25), anti-MED12 (BethylLaboratories A300-774A; 1:100) or anti-pSTAT1^ser727^ (ThermoFisher MA5-38231; 1:50). Sections were 0.1% TBS-Tween20 washed three times for 10 min before incubation with Goat anti-Rabbit HRP (Agilent P0448; 1:200) for 1-h (room temperature), followed by three further 10-min washes. Antibody staining was revealed using Signalstain® DAB Substrate Kit (Cell Signalling Technologies) for 3 min.

Central sections from STR/Ort right knees from DMSO (*n* = 5) and MSC-818-treated (*n* = 6) mice were processed according to the above and probed with anti-Chondromodulin-1 (AffinityBioscience Ltd DF15727-AFF; 1:200) or anti-Matrillin-3 (ThermoFisher PA5-112604; 1:2 000).

### CDK8 localisation and expression in ATDC5 micromasses

5.4 × 10^4^ ATDC5 micromasses were seeded on black-walled glass 96-well plates (Greiner SensoPlate) and differentiated for either 7 days in differentiation media before treatment with IL1β for 24-h, or 8 days in differentiation media only as previously described above (*n* = 5). Micromasses were then fixed with 4% PFA for 5 min before permeabilisation with 0.1% TBST for 15 min at room temperature. Micromasses were subsequently incubated with blocking buffer (10% Goat serum, 0.1% bovine serum albumin TBS) for 30 min, before overnight incubation at 4 °C with anti-CDK8 (Invitrogen PA5-115001; 1:100). Wells were then washed in 0.1% TBS-Tween20 before incubation with goat anti-Rabbit IgG (AlexaFluor 488 Invitrogen A11008; 1:500) for 30 min at room temperature. Nuclei were stained with Hoechst 33342 (5 µg/mL, Invitrogen) for 10 min at room temperature before washing in 0.1% TBS-Tween20. Cells were then mounted in fluorescent mounting media (DAKO, Agilent) 30 min prior to confocal imaging using a Leica TCS SP8 laser scanning confocal microscope. To quantify, the relative fluorescence units (RFU) for each entire well (5 replicates per condition) was calculated using manufacturers software after a whole well fluorescence scan at 488 nm wavelength (SparkCyto Tecan) with the well average RFU plotted.

### Statistical analysis

For statistical analysis of two groups (i.e. control vs. drug-treated), *t*-test with Welch correction was conducted. For the analysis of >2 groups, two-way ANOVA (specific post-hoc test for multiple comparisons highlighted within individual figure legends) was performed. A paired *t*-test was conducted for statistical analysis of parametric data, whilst Wilcoxon matched pairs used for nonparametric data for the same sample under different conditions. Mann-Whitney test was used for non-normal distributed and/or ordinal data of vehicle- vs drug-treated conditions. Each figure legend details the specific statistical method used to identify significance for the corresponding figure. Data are presented as either fold change (FC) or percentage change, depending on experimental approach.

## Supplementary information

Supplementary File 1

Supplementary File 2

## Data Availability

All the data support the figures, and the other findings are available upon reasonable request to the corresponding authors. Supplementary information accompanies the manuscript on the Bone Research website.
