# Supplementary material for: Inhibition of cyclin-dependent kinase 8 modulates chondrocyte transition to hypertrophy and provides in vivo protection against spontaneous osteoarthritis
Source: Bone Res. 2026 Aug 25;14:89. doi: 10.1038/s41413-026-00551-3 (PMC13503767; doi:10.1038/s41413-026-00551-3)
Supplement: Supplementary file 1 [file 41413_2026_551_MOESM1_ESM.docx]

**Methods**

**Small molecule screen on ATDC5 micromasses**

An epigenetic library consisting of 226 known epigenetic/transcriptional targeting compounds (kind gift from Prof. Oppermann, University of Oxford) was used in a phenotypic screen with ATDC5 high density micromasses to identify any compounds that have an inhibitory effect on matrix mineralisation (catabolic chondrocyte) whilst maintaining and/or promoting healthy extracellular matrix deposition (anabolic chondrocyte)

The developed screening method miniaturises the classic culture conditions, whilst shortening the culture period compared to standard published chondrogenic assays offering a novel method for compound screening of chondrogenic cultures (1).

In the developed screening method, 5.4x10^4^ cells in a total volume of 2µl standard growth media (DMEM-F12 (Gibco), 5% FBS, 1% Ab/Am (Gibco) 10µg/ml human transferrin (Sigma), 3x10^-8^M sodium selenite (Sigma) were seeded onto single wells of a 96-well plate and incubated at 37°C, 5% CO_2_ in a humidified incubator for 2 hours to allow for cell interactions to form. 100µl of differentiation media (DMEM-F12, 5% FBS, 1% Ab/Am, 5µg/ml human transferrin, 1 x ITS (Gibco)) containing a single compound was added to each well.

Plates were seeded in duplicate to observe the effect each compound had on glycosaminoglycan (GAG) and mineral deposition at the end of the culture. Chondrogenesis of the cultures was promoted in differentiation media in the presence of the compounds for 7 days with media changes containing library every 2-3 days. On day 7, media was switched to a mineralisation media (Alpha-MEM (Gibco), 5% FBS, 1% Ab/Am, 7mM β-Glycerophosphate, 50µg/ml ascorbic acid, 5µg/ml human transferrin, 1x Insulin-Transferrin-Selenium premix) and again supplemented with compounds for 7 days with media changes every 2-3 days.

On day 14, culture plates were fixed with 95% ice-cold methanol for 25 minutes at -20˚ C and each compound-treated well stained with either Alcian blue 8GX (Alfa Aesar; 0.5% w/v, 1M HCl; 25 minutes before washing with dH_2_O) or Alizarin red (1% w/v dH_2_O, 5 minutes before washing with 50% EtOH). For quantification, Alcian blue-stained wells were leached via incubation with 8M Guanidine HCl (Sigma) at 37˚ C with gentle agitation for up to 6 hours before absorbance measurement at 650nm. Alizarin red stained wells were incubated with 10% Cetylpyridinium chloride (CPC; Sigma) at 37˚ C with gentle agitation for 1 hour and absorbance measured at 570nm.

To identify hits, the Alcian blue:Alizarin red absorbance ratio was plotted and readings higher than the control group ratios were classified as hits. To prevent data skewing, Alcian blue absorbances below 60% of the control mean were set to 0 to identify compounds that maintain/promote healthy extracellular matrix production and reduced mineralisation simultaneously. Hits were cross-referenced with images of stained micromasses to confirm reduction in Alizarin red and maintenance/enhancement of Alcian blue and selected hits were subjected to dose response assays and gene expression analysis. Briefly, 5.4x10^4^ ATDC5 cells were seeded in 2µl micromasses and differentiated in the presence of epigenetic compounds (ACBI1, SGI-1776, AZD5153, MZ1, BI1347, JQ1(+) and CI1994) at serial diluted concentrations ranging 10µM – 1pM or DMSO control. This was repeated for Flavopiridol, a broad spectrum CDK inhibitor to determine whether the effects observed were CDK8/19 specific. For gene expression analysis, 2.7x10^5^ ATDC5 cells were cultured in 10µl micromasses in the presence of selected epigenetic compounds (700nM ACBI1, 500nM MZ1 or 1µM BI1347). On day 7 and 14 micromasses were processed for extracellular matrix (ECM) staining and gene expression analysis via qPCR. For qPCR analysis, RNA was extracted from micromasses using the Reliaprep miniprep RNA extraction kit (Promega) before conversion to cDNA using the High-capacity reverse transcription kit (Applied Biosciences). qPCR was performed using qPCRBIO SyGreen® Blue Mix Lo-ROX (PCR Biosystems) with data acquisition using BioRad CFX96.

**Intracellular ROS and SO- quantification**

5.4x10^5^ ATDC5 cells were seeded in a 2µl micromass on a 96-well plate in triplicate per condition. Some micromasses were differentiated for 9 days in ATDC5 differentiation media (chondrogenic conditions) whilst others were differentiated for 7 days in ATDC5 differentiation media before switching to ATDC5 mineralisation media (induced hypertrophy/mineralisation) or ATDC5 IL1β media for 48-hours. For all conditions cells were either treated with DMSO or 100nM MSC2530818 (MSC-818) for the entire culture period. On day 9, ROS/SO^-^ quantification was carried out using ROS/SO^-^ assay (Abcam). Briefly, some cells were pretreated with 5mM N-acetyl cysteine (ROS inhibitor) for 30 minutes prior to the assay. ROS/SO^-^ detection media (1µM oxidative stress detector, 1µM superoxide detector DMEM F12) was added to cells, with some wells co-treated with 200µM pyocyanin (ROS inducer) for 30 minutes as per manufacturer instructions. Cells were then washed 3 times in PBS before fluorescent intensity measured at Ex/Em 490/525nm for total ROS and Ex/Em 550/620nm for SO^-^ (Tecan SparkCyto)

**Nitric oxide quantification**

10µl ATDC5 micromasses were differentiated as previously described. On day 9, culture media was subjected to nitric oxide colorimetric assay (CheKine, Abbkine), which uses an improved Greiss method to quantify the reduction of nitrate to nitrite. Nitrites are rapidly decomposed into nitric oxide; thus, this is an indirect measure of nitrite production. Culture media and NaNO_2_ standard curve samples were deproteinated via the addition of ZnSO_4_ and centrifuged for 10 minutes at 14,000xg at 4˚ C. 100µl of deproteinated samples/standards were added to freshly prepared working reagent (100µl VCl_3_, 50µl Greiss Reagent l, 50µl Greiss Reagent ll per sample) and incubated at 37˚ C for 30 mins. Absorbance was measured at 540nm, and samples were blank-corrected before calculation of nitric oxide quantification from the standard curve.

**SOD dismutase activity quantification**

SOD dismutase is a potent antioxidant which catalyses the conversion of superoxide anion (O_2_^-^) into hydrogen peroxide (H_2_O_2_), reducing cellular damage (2). SOD dismutase activity analysis was carried out via the superoxide dismutase (SOD) activity assay kit (CheKine), which provides O_2_^-^ that reacts with tertrazolium salt WST-8 dye within the working reagent to form a coloured formazan product which can be measured at 450nm. In the presence of SOD, O_2_^-^ is scavenged and therefore less is available for the reaction to form the coloured formazan product. Therefore, the greater the absorbance measured the lower the SOD activity (3).

**Primary osteoclast isolation and differentiation**

Primary bone marrow mononuclear cells were isolated from the bone marrow of the long bones of 4, 8-week-old C57/BL-6 mice. Briefly, all soft tissue was removed from the long bones before the epiphyses was cut and the bone marrow flushed using a 25-gauge needle and PBS. The extracted bone marrow solution was then centrifuged at 377xg for 5 minutes before the pellet was resuspended in osteoclast basal media (MEM supplemented with 10% FBS, 2mM L-glutamine, 1% Ab/Am) for 24 hours. Note, all media used for osteoclast experiments were phenol free. The cell suspension was then removed, centrifuged at 377xg and resuspended in osteoclast differentiation media (basal media supplemented with 100nM PGE_2_, 200ng/ml MCSF, 3ng/ml RANKL pH 7.3), before 1x10^6^ cells in 200µl media were seeded onto dentine discs placed within each well of a 96-well plate for 24 hours. Discs were then transferred to 6-well plates (maximum 5 discs per well) in 3ml osteoclast differentiation media containing 1µM BI1347 or DMSO for 3 days (N=8). On day 5 of culture, osteoclast resorption was initiated in the presence of 1µM BI1347 by addition of 12M hydrochloric acid to acidify differentiation media to pH 7.0 for 2 days. On day 7, discs were fixed in 2.5% glutaraldehyde for 5 minutes before washing in PBS. Discs were then stained with 2.5ml tartrate resistant acid phosphatase (TRAP) staining solution per well using the leukocyte acid phosphatase kit (Sigma) as per manufacturer instructions.

For image analysis, two images per disc were acquired using the 5x objective (HC PL FLUOTAR, NA=0.15) with reflective light using the DM400B upright microscope with a DFC550 colour camera through the Leica application suite/ LAS-X v3.7 (Leica Microsystems). Acquired images were subjected to the automated counting method which implements an algorithm to count pre-osteoclast and osteoclast number. This method was developed by Davies *et al*. 2023 using Ilastik software in Image J (Fiji, USA) (4). Resorption area on each disc was manually counted and analysed using Image J software. Briefly, TRAP-positive cells were identified as osteoclasts with clear evidence of resorption pit formation surrounding the cell or ≥2 nuclei. To quantify resorption area, a 16x12 grid (0.08 inches^2^ per point) was overlaid on the image. The total area resorbed was calculated using the known area of the dentine disc, the acquired image and the grid size. The sum of the two images per disc were taken and results are expressed as pre-osteoclast/osteoclast number and total area resorbed (mm^2^)(5).

**MC3T3 osteoblast-like cell differentiation**

MC3T3 (subclone 14) osteoblast-like cells were expanded in growth media (Alpha-MEM supplemented with 10% FBS, 1% Ab/Am). Cells were then seeded on a 24-well plate at a density of 2x10^4^ and cultured for 2 days in osteoblast growth media before induction of differentiation with osteoblast differentiation media (Alpha-MEM, 10% FBS, 1%Ab/Am, 50µg/ml ascorbic acid, 5mM β-glycerophosphate) in the presence of 1µM BI1347 or DMSO, with media changes every 2-3 days. A further optimised MC3T3 differentiation method was also carried out with MSC-818 treatment. Briefly, MC3T3 cells were seeded as above and differentiated for 7 days in osteogenic induction media (Alpha-MEM, 10% FBS, 1%Ab/Am, 50µg/ml ascorbic acid) with DMSO or 100nM MSC-818, before switching to osteogenic induction media with 2mM β-glycerophosphate in the presence of 100nM MSC-818 or DMSO. Cells were fixed in 5% PFA for 10 minutes before washing in PBS and air drying. Cells were then stained with 1% Alizarin Red on day 14. Alizarin red stain was leached and quantified as previously described.

**MLO-A5 post-osteoblast to pre-osteocyte differentiation with CDK8i**

MLO-A5 cells were expanded in osteocyte growth media (5% heat inactivated FBS, 5% heat inactivated calf serum, 1% ab/am, Alpha-MEM) before seeding at 3.5x10^4^/cm^2^ in osteocyte growth media for 2 days. Media was then switched to osteocyte mineralisation media (10% FBS, 100µg/ml L-Ascorbic acid 2-phosphate, 4mM β-glycerophosphate, 1% ab/am, Alpha-MEM) containing either DMSO, 100nM BI1347 or 100nM MSC-818 for 7 days. Media was changed every 2-3 days with fresh compound, L-Ascorbic acid 2-phosphate and β-glycerophosphate added each time. On day 7 cells were fixed with 4% PFA before staining with Alizarin red and quantification via CPC.

**Results**

**Epigenetic compound library contains modulators that reduce mineralisation in ATDC5 and maintain matrix deposition?**

Differentiating ATDC5 micromasses in the presence of epigenetic compounds displayed notable differences in the effects of epigenetic modulators on mineralisation (Alizarin red stain) and GAG deposition (Alcian blue stain) across the library (**supplementary figure 1**). Stains were leached for quantification and a staining ratio plotted (Alizarin red Absorbance/Alcian blue absorbance) to observe compounds that reduce mineralisation but do not negatively affect GAG deposition, thus highlighting compounds that inhibit aspects of chondrocyte hypertrophy such as matrix mineralisation. Several hits were identified: ACBI1, BI1347, MZ1, AZD5153, Bromosporin, SGI-1776, PFI-1, CHR-6949, ZXH-3-26 and CI-994, which displayed a greater ratio than the DMSO control threshold (defined by the highest and lowest measurements of the DMSO control readings within each plate). When compounds were added on day 7 of culture, no differences in staining were observed compared to untreated control, suggesting inhibitors need to be added earlier on to reveal modulators of chondrocyte hypertrophy in this screening platform.

**Supplementary figure 1. Epigenetic compound library screen using a miniaturised ATDC5 screening platform**. **A)** Example of Alizarin red staining (mineralisation) and Alcian blue staining (glycosaminoglycan) from a library plate with compound addition from day 0 of culture. **B)** Graphs of Alcian blue:Alizarin red absorbance ratio from the epigenetic library screen (N=1). ACBI1, BI1347, MZ1, AZD5153, Bromosporin, SGI-1776, PFI-1, CHR-6949, ZXH-3-26 and CI-994 identified as hits with absorbance ratios greater than the DMSO control threshold. Dotted lines indicate DMSO control window.

**Dose response of epigenetic library hits**

Commercially available molecules (ACBI1, AZD5153, BI1347, MZ1, SGI-1776 and CI-1994) and their negative controls (where available) were used in a dose response assay to determine optimal concentrations that would preserve Alcian blue staining whilst having an inhibitory effect on Alizarin red staining. Interestingly, BI1347 did not display any negative effects on GAG deposition, as a constant Alcian blue absorbance of 0.4_Abs650nm_ was maintained at all concentrations, whilst at 1µM Alizarin red absorbance was reduced by 40%. JQ1(+) reduced both stains at concentrations in the picomolar range with detrimental effects on GAG deposition seen at higher concentrations, so was not taken forward for further investigation. ACBI1 and MZ1 show identical trends in their dose response effects with reduction in Alizarin red staining observed at 0.5µM that preserves Alcian blue within the control window. AZD5153 reduces Alizarin red staining in a dose dependant manner up to 0.01µM where absorbance readings plateau, at this concentration Alcian blue absorbances drop below the threshold (**supplementary figure 2**).

**Supplementary figure 2. Dose response of hits from epigenetic screen.** Log_10_ absorbance values at 570nm (Alizarin red) and 650nm (Alcian blue) of dose response assay on ATDC5 micromasses fixed and stained on day 14. Compound added on day 0 of culture starting at 10µM and titrated 10-fold down to 1pM. Pink box indicates Alizarin red DMSO control; light blue box indicates Alcian blue DMSO control. (Log(inhibitor) vs. Response-variable slope (four parameter) fit) (N=1).

**Identification of BI1347 (CDK8i) as candidate for further investigation**

BI1347, MZ1 and ACBI1 were taken forward for day 7 (chondrogenic; *SOX9, ACAN, COL2A1*) and day 14 (hypertrophic; *COL10A1*^(315)^) gene expression analysis (**supplementary figure 3**). Out of the three compound treatments, only BI1347 displayed a favourable gene expression profile representative of enhanced chondrogenic and supressed hypertrophic gene expression. Day 7 gene expression analysis found BI1347 treatment to enhance *COL2A1* and *ACAN* expression (FC: 4.8 and 6, respectively). On day 14, BI1347 treatment increased *SOX9* (FC: 1.8) and *ACAN* (FC: 6), whilst reducing *COL10A1* expression (FC: 0.03). ACBI1 and MZ1 treatment notably reduced all chondrogenic gene expression investigated on day 7 and 14 indicating that these compounds may supress chondrogenesis and therefore unfavourable candidates. From this point forward BI1347 was selected for further investigation.

**Supplementary figure 3. Gene expression analysis of Day 7 and 14 ATDC5 micromasses following treatment with epigenetic hits.** Treatment with 1µM BI1347 (CDK8i) displays a significant increase in gene expression of cartilage ECM components COL2A1 and ACAN on day 7 and 14, in addition to increasing SOX9 expression (day 14 only), whilst suppressing COL10A1 expression under hypertrophic conditions on day 14 compared to DMSO control. MZ1 and ACBI1 displayed no effect/reduction on gene expression across timepoints. Statistical significance from DMSO control determined via two-way ANOVA (Šídák's multiple comparison test), *p≤0.05, **p≤0.01, ***p≤0.001, ****p≤0.0001 (N=3)).

***Supplementary figure 4. Enrichment analysis of upregulated differentially expressed genes from ATDC5*** ***RNAseq****. Biological* *and KEGG pathway analysis of day 7 upregulated DEGs. Note cartilage development as the top enriched biological pathway, whilst KEGG analysis identified GAG biosynthesis as the top enriched pathway. Bar colour represents log10(FDR) with yellow= highest, blue= lowest. Size of dot represents number of genes.*

**Supplementary figure 5. Top 10 upregulated and downregulated protein encoding genes from ATDC5** **RNAseq**. Table of 10 most upregulated DEGs (top) shows a significant increase in known chondroprotective genes (TIMP4 (Log2 Fold 6.85) and Chondromodulin-1 (Log2 Fold 6.81)). Table of 10 most downregulated DEGs (bottom) shows a significant decrease in inflammatory mediators related to cartilage destruction such as CXCL11 (Log2 Fold -7.89).

**Supplementary figure 6. ATDC5** **RNA-Seq selected DEGs of interest.** Tables of selected interesting DEGs in both the upregulated (Top) and downregulated (Bottom) data sets including the name of the protein encoded by the gene and documented function in relation to cartilage health and disease.

***Supplementary figure 7.*** *Chemical structures of CDK8i tested on ATDC5 micromasses*

**Chondrogenic effect is specific to CDK8i**

To determine whether the effect observed on ATDC5 micromasses was specific to CDK8i or whether this was a broad-spectrum effect of inhibition of CDKs, a dose response assay treating ATDC5 micromasses with Flavopiridol (throughout differentiation, as previously described) was carried out. As shown in **supplementary figure 8**, non-specific CDK inhibition does not induce a pro-anabolic phenotype in ATDC5 micromasses with a trend for reduced GAG deposition at 1 µM. At 100 nm, there is a trend for increased GAG deposition and trend for reduced decrease in mineralisation, however this is minimal and does not produce the same trends as with CDK8i.

***Supplementary figure 8. Dose response of broad spectrum CDK inhibitor Flavopiridol.*** *Alizarin red (mineralisation; N=1) and Alcian blue (GAG; N=2) quantification of day 14 ATDC5 micromasses treated with a dose response of broad spectrum CDK inhibitor Flavopiridol.*

**CDK8i supresses osteoblast-mediated mineralisation, osteoclast-mediated resorption and promotes osteocyte maturation in vitro**

To determine the effects of CDK8i on osteoblast differentiation, MC3T3 osteoblast-like cells were differentiated in the presence of 100nM MSC-818. Treatment with MSC-818 ablated matrix mineralisation, whilst DMSO control conditions displayed robust matrix mineralisation by day 14, suggesting that CDK8i may impede osteoblast maturation and/or mineralisation processes. Differentiating C57/BL-6 murine bone marrow derived cells towards osteoclasts in the presence of 1 µM BI1347 showed no effect on pre-osteoclast or mature osteoclast numbers compared to DMSO control. However, BI1347 treatment significantly reduced osteoclast resorption (FC: 0.43), suggesting CDK8 may play a role in osteoclastic activity. This is particularly important as increased osteoclast resorptive activity has been found to correlate with disease progression in OA. MLO-A5 cells are a pre-osteocyte cell line used as a model of post-osteoblast to pre-osteocyte differentiation (6). As CDK8i evidently displayed an inhibitory effect on osteoblast driven matrix mineralisation potentially by affecting osteoblastogenesis, the effects of CDK8i on osteoblast-osteocyte differentiation was explored. Matrix mineralisation was present in all conditions on day 7, with a significant increase (FC: 1.3) in the 100 nM MSC-818 condition. Indeed, interrogation of the osteocyte transcriptome with CDK8i would provide a better insight into the mechanisms behind the increased mineralisation observed (**supplementary figure 9**).

***Supplementary figure 9. CDK8i effect on bone cells. (a)*** *Day 14 Alizarin red stained and quantified MC3T3 osteoblast-like cells treated with 100nM MSC-818. Note treatment permits matrix deposition however ablates mineralisation. Scale bar= 100µm, N=3, error bars= SD, Welch’s T-test.* ***(b)*** *C57/BL-6 bone marrow cells differentiated towards osteoclasts in the presence of 1µM BI1347 and TRAP stained show no change in pre-osteoclast or mature osteoclast number compared to DMSO control, however, a reduction in osteoclast resorptive activity is observed (N=8, scale bar 200µm, error bars = SD, Welch’s T-test).* ***(c)*** *Alizarin red stained and quantified MLOA5 pre-osteocyte cells differentiated towards osteocytes in the presence of DMSO, 100nM BI1347 and 100nM MSC-818. Scale bar= 100 µm. (N=3, error bars = SD, statistical analysis = two-way ANOVA Dunnet’s multiple comparison, * p≤0.05).*

**CDK8i modulates intra and extracellular ROS/RNS production by ATDC5 micromasses**

To determine whether CDK8i modulates oxidative stress in chondrocytes, ROS, SO^‑^ and NO^-^_2_ oxidant species were quantified in ATDC5 micromasses. As shown in **supplementary figure 10**, nitrite concentration was low in both chondrogenic and hypertrophy/mineralisation conditions in ATDC5 micromasses across DMSO and MSC-818 treatment groups. IL1β treatment robustly increased nitrite production by the cells validating the use of IL1β to induce nitric oxide production in ATDC5 cells. In this model, MSC-818 treatment reduced NO^-^_2_ in ATDC5 micromasses (FC: 0.8). Analysis of SOD dismutase (a potent antioxidant savaging superoxide anions) suggests MSC-818 may increase SOD activity under chondrogenic conditions, potentially as a result of increased metabolic activity. SOD activity is stabilised in the DMSO group across chondrogenic and IL1β treatment, however MSC-818 appears to reduce SOD activity (FC: 0.4) in IL1β conditions. This is likely because of less RNS/ROS with MSC-818 treatment meaning less SOD is required to neutralise.

***Supplementary figure 10. ROS, SO^-^, NO_2_^-^ and SOD dismutase quantification of MSC-818 treated ATDC5 micromasses****.* ***(a)*** *Intracellular ROS quantification* ***(b)*** *SOD dismutase* ***(c)*** *intracellular SO* ***(d)*** *NO_2_^-^ quantification in ATDC5 micromasses under chondrogenic, mineralisation (a,c,d only) and IL1β (OA-like) conditions following 100nM MSC-818 treatment . a,c,d Statistical analysis = Šídák's ANOVA, error bars= SD, **p=≤0.01, N=3. b N=1.*

**CDK8i has no effect on OARSI score**

OARSI scoring of all four condyles of both left and right limbs of CDK8i treated STR/Ort mice found no differences in condyle average or max condyle average score (**supplementary figure 11**).

***Supplementary figure 11. OARSI scoring of CDK8i treated Str/Ort mice.*** *Pooled left and right limb scores for each condyle where possible. No significant differences in the average condyle or max condyle average score with MSC-818 treatment. MT; medial tibia, LT; lateral tibia, MF; medial femur and LF; lateral femur. Error bars = SD, statistical significance= 2-way ANOVA.*

***Supplementary figure 12. Chondrocyte pericellular matrix scoring system.*** *Scoring system devised from RGB stained Str/Ort and CBA tibial articular cartilage sections. Note healthy pericellular matrix in abundant in proteoglycan and has a score of 1, whilst disease increases in score based off severity of proteoglycan loss.*

***Supplementary figure 13. Bone matrix scoring system.*** *Scoring system devised based on Masson’s Trichrome staining on the tibial subchondral bone of CBA and Str/Ort knee sections. Note healthy subchondral bone exhibits little if any remodelling thus scores are 0, whilst remodelling processes increase with disease severity and score.*

**References**

1.	Castaño Betancourt MC, Cailotto F, Kerkhof HJ, Cornelis FM, Doherty SA, Hart DJ, et al. Genome-wide association and functional studies identify the DOT1L gene to be involved in cartilage thickness and hip osteoarthritis. Proc Natl Acad Sci U S A. 2012;109(21):8218-23.

2.	Younus H. Therapeutic potentials of superoxide dismutase. Int J Health Sci (Qassim). 2018;12(3):88-93.

3.	Weydert CJ, Cullen JJ. Measurement of superoxide dismutase, catalase and glutathione peroxidase in cultured cells and tissue. Nat Protoc. 2010;5(1):51-66.

4.	Davies BK, Hibbert AP, Roberts SJ, Roberts HC, Tickner JC, Holdsworth G, et al. A Machine Learning-Based Image Segmentation Method to Quantify In Vitro Osteoclast Culture Endpoints. Calcif Tissue Int. 2023;113(4):437-48.

5.	Orriss IR, Arnett TR. Rodent osteoclast cultures. Methods Mol Biol. 2012;816:103-17.

6.	Kato Y, Boskey A, Spevak L, Dallas M, Hori M, Bonewald LF. Establishment of an osteoid preosteocyte-like cell MLO-A5 that spontaneously mineralizes in culture. J Bone Miner Res. 2001;16(9):1622-33.
